# Calves Infected with Virulent and Attenuated *Mycoplasma bovis* Strains Have Upregulated Th17 Inflammatory and Th1 Protective Responses, Respectively

**DOI:** 10.3390/genes10090656

**Published:** 2019-08-28

**Authors:** Jin Chao, Xiaoxiao Han, Kai Liu, Qingni Li, Qingjie Peng, Siyi Lu, Gang Zhao, Xifang Zhu, Guyue Hu, Yaqi Dong, Changmin Hu, Yingyu Chen, Jianguo Chen, Farhan Anwar Khan, Huanchun Chen, Aizhen Guo

**Affiliations:** 1 The State Key Laboratory of Agricultural Microbiology, Huazhong Agricultural University, Wuhan 430070, China; 2 College of Veterinary Medicine, Huazhong Agricultural University, Wuhan 430070, China; 3 Hubei International Scientific and Technological Cooperation Base of Veterinary Epidemiology, Huazhong Agricultural University, Wuhan 430070, China; 4 Key Laboratory of Development of Veterinary Diagnostic Products, Ministry of Agriculture, Huazhong Agricultural University, Wuhan 430070, China; 5 Wuhan Keqian Biology Ltd., Wuhan 430223, China

**Keywords:** cattle, *Mycoplasma bovis*, pathogenesis, peripheral blood mononuclear cells, transcriptome, Th17, Th1

## Abstract

*Mycoplasma bovis* is a critical bovine pathogen, but its pathogenesis remains poorly understood. Here, the virulent HB0801 (P1) and attenuated HB0801-P150 (P150) strains of *M. bovis* were used to explore the potential pathogenesis and effect of induced immunity from calves’ differential transcriptomes post infection. Nine one-month-old male calves were infected with P1, P150, or mock-infected with medium and euthanized at 60 days post-infection. Calves in P1 group exhibited other clinical signs and pathological changes compared to the other two groups. Transcriptome profiles of peripheral blood mononuclear cells revealed seven and 10 hub differentially expressed genes (DEGs) in P1 and P150 groups compared with mock-infected group, respectively. Then, P1-induced pathogenesis was predicted to be associated with enhanced Th17, and P150-induced immunity with Th1 response and expression of ubiquitination-associated enzymes. Association analysis showed that 14 and 11 DEGs were positively and negatively correlated with pathological changes, respectively. Furthermore, up-regulated expression in molecules critical to differentiation of pathogenic Th17 cells in lung and peripheral blood mononuclear cells in P1 group was validated at RNA and protein levels. The results confirmed virulent and attenuated strains might be associated with biased differentiation of pro-inflammatory pathogenic Th17 and Th1 subsets respectively.

## 1. Introduction

Mycoplasmas are the smallest self-replicating and cell wall-less organisms. Among them, *Mycoplasma bovis* is an important pathogen infecting bovines worldwide by primarily causing pneumonia, as well as other disorders such as mastitis, arthritis, and otitis. Some characteristics related to the pathogenesis of *M. bovis* were confirmed in vivo, such as colonizing the mucosal surfaces, invading tissues, persisting at the site of infection [1], inhibiting the respiratory burst of neutrophils [2,3], and inducing T-helper (Th) 2-biased response [1]. Furthermore, *M. bovis* has been reported to escape the specific immune response of hosts by employing the strategies such as variation in surface antigens, inappropriate activation of alveolar macrophages, recruitment of neutrophils to the inflammation sites [4,5], release of inflammatory mediators like interleukin (IL) IL-6, TNF-α, IL-1β [6], and IL-10 [7], etc. Additionally, some controversial conclusions remain on whether *M. bovis* induces apoptosis in bovine monocytes, lymphocytes, and neutrophils in vitro [7,8,9]. Despite these advances in the understanding of *M. bovis* pathogenesis, important knowledge gaps remain.

The Th17 immune response is characterized by IL-17 production. So far, contradictory conclusions have been obtained by different investigators regarding to the functions of Th17 immune responses induced by various mycoplasma species. Some studies have reported that IL-17 is essential for defense against *Mycoplasma pulmonis* [10], *Mycoplasma pneumoniae* [11], and a secondary *Listeria* infection [10]. However, others demonstrated that *M. pulmonis* induced Th17 response associated with pathogenesis, as shown by the observation that neutralization of IL-17A with anti-IL-17A antibodies reduced disease severity and the incidence of neutrophilic lung lesions during *M. pulmonis* infection in mice [12]. Recently, virulent *M. bovis* has been reported to induce IL-17 production in the peripheral blood mononuclear cells (PBMCs) of calves [13], whereas the recombinant bovine IL-17A could not enhance the capacity of neutrophils to destroy *M. bovis* in vitro [8]. A partial explanation for these paradoxical results is that the authors failed to consider the different functions of protective and pro-inflammatory pathogenic Th17 subset cells, whose differentiation depends on the host microenvironment. Therefore, the aforementioned reports suggest the necessity of further confirmatory studies on whether the response of Th17 subsets of cattle immune cells during *M. bovis* infection in vivo plays either a protective or pathogenic role in cattle.

Previously, an attenuated Chinese strain *M. bovis* HB0801-P150 derived from its wild-type *M. bovis* HB0801 strain [14] was demonstrated to protect cattle against virulent challenge [15]. Thus, these two strains can serve as ideal materials to investigate the factors of *M. bovis* pathogenesis. Furthermore, PBMCs are clinically relevant cells with well-established roles in host immune responses to microbial pathogens [16]. The objective of this study was to reveal the underlying mechanism for pathogenesis of virulent P1 strain and immunity of attenuated P150 strain by performing a comparative transcriptome analysis of PBMCs from calves infected with both strains and confirming the transcriptome findings using the data from the blood and lung tissues and gross pathology of the experimental cattle. These in vivo results facilitated the understanding of *M. bovis*–induced immunity, which will contribute to the development of measures against *M. bovis* infection in cattle. 

## 2. Materials and Methods 

### 2.1. Ethics Statement

The animals were maintained and the experiments conducted on the experimental farm of Huazhong Agricultural University, Wuhan, China. This study was approved on 21 October 2015 by the Ethics Committee of Huazhong Agricultural University (#SYXK(er) 2015-0084) in accordance with the university ethical guidelines for scientific research as well as the Ethical and Legal Principles of the Hubei Regulations for the Administration of Affairs Concerning Experimental Animals.

### 2.2. Mycoplasma Bovis Strains and Culture

The attenuated *M. bovis* HB0801-P150 strain stored in the China Center of Type Culture Collection (Wuhan) (CCTCC No.: M2011102) was obtained after continuous cultivation for 150 passages in vitro. Its wild-type *M. bovis* HB0801 (CCTCC No.: M2010040) was isolated from infected cattle [14]. Both the strains were cultured in complete pleuropneumonia-like organism (PPLO) medium as described previously [15]). The complete PPLO medium was inoculated with *M. bovis* strains at a ratio of 1:1000 and cultured at 37 °C in 5% CO_2_ for approximately 48 h. The concentration of *M. bovis* was calculated as colony forming units (CFU) per mL as previously described [15]. Briefly, the culture of each isolate was serially 10-fold diluted in phosphate buffered saline (PBS), in triplicate. Then, each dilution was plated on PPLO agar and incubated at 37 °C in 5% CO_2_. After three days, the colonies were counted with a stereomicroscope, and the concentration of CFU/mL was expressed as the mean. After infection, the titers of diluted inoculum were confirmed with the same method.

### 2.3. Calf Infection with M. bovis Strains

Nine one-month-old male calves were purchased from a local dairy farm (Wuhan, China) under the following criteria: clinically healthy, negative to *Mycoplasma* infection as defined by negative nasal swabs to *Mycoplasma* culture isolation and PCR of 16S rRNA; negative to common respiratory pathogens shed in nasal cavity; negative serum antibody against *M. bovis* was determined using a commercially available *M. bovis* antibody ELISA kit (#BIO K 302, Bio-X Diagnostics S.A., Brussels, Belgium) according to the manufacturer’s instructions. Furthermore, the antibody of other common pathogens, including bovine herpesvirus 1 (BoHV-1) and bovine viral diarrhea virus (BVDV), was tested using commercially available ELISA kits (IDEXX IBR gE Ab test, #99-41299 and BVDV total Ab test, #99-44000; IDEXX, USA). Subsequently, the calves were randomly divided into three groups (*n =* 3 each)—P1 (HB0801), P150, and mock-infected groups. Each calf in the P1 and P150 groups was nasally inoculated with about 10^9^ CFU of either HB0801 or P150 strain in 5 mL of PPLO broth (2 × 10^8^ CFU/mL). The mock-infected calves were mock-inoculated with an equal volume of sterile PPLO medium broth. The animals in different groups were separately housed in individual rooms. At the end of the study period (60 days post-infection), all the calves were euthanized for necropsy. After pathological examination post mortem, the bodies and related wastes were collected and incinerated by the local professional processing plant for dead animals. Additionally, the pleural fluid, joint fluid, trachea swabs, hilar lymph nodes, and lung tissues at the diseased and healthy border were collected to isolate *M. bovis*. The lung tissue samples were fixed, embedded, sectioned [14], and stained with hematoxylin and eosin (H&E) by conventional methods, followed by observation using a microscope (BX53, Olympus, Tokyo, Japan). 

### 2.4. Clinical Observation and Sample Collection

Clinical observation was performed daily for 60 days. The rectal temperature was measured and recorded between 07:30 and 09:00 A.M. before morning feeding. The general clinical signs such as fever, anorexia, sluggishness, nasal discharge, severity and duration of cough, and joint swelling were recorded. The calves were weighed immediately before infection (day 0) and euthanasia (day 60). On 0, 1, 3, 5, 7, 12, 15, 18, and 22 days post-infection (dpi), nasal swabs were obtained immediately after temperature measurement and collected separately in tubes containing 2 mL of sterile normal saline to isolate *M. bovis* within 8 h. Ten microliters of heparinized blood with heparin sodium was collected at 0, 7, and 14 dpi to isolate PBMCs. The whole blood was also sampled at 0, 1, 7, 14, 28, 35, and 60 dpi to detect the serum antibody of *M. bovis* by using a commercially available *M. bovis* antibody ELISA kit mentioned above.

When typical clinical signs of *M. bovis* infection occurred in calves from P1 compared to P150 and mock-infected groups, PBMCs from all calves were isolated and subjected to RNA microarray assay as described below, and the remaining PBMCs were used to confirm the findings of the microarray assay by quantitative PCR (qPCR).

### 2.5. Detection of Potential Pathogens from Swabs, Tissues and Fluids

After violent agitation, the nasal and tracheal swabs (to isolate *M. bovis* in the tracheal mucus) in the tubes were removed, and the supernatants were passed through 0.45-μm filters to exclude debris and contaminating bacteria. Subsequently, pre-filtered samples were incubated with PPLO broth and agar at 37 °C in 5% CO_2_ for approximately 3 to 7 days. As for tissues, 0.5 g of tissue samples was homogenized with 2 mL of sterile normal saline beforehand. The homogenate of tissues and fluids sampled were centrifuged at 1000× *g* for 5 min at 4 °C, and then the supernatants were passed through 0.45-μm filters and incubated with PPLO broth and agar as above to isolate *M. bovis*. 

The PCR of *M. bovis* uvrC gene was performed to distinguish *M. bovis* isolated from nasal and tracheal swabs as well as tissues from other Mycoplasma species as described previously using the 238 bp PCR products of *M. bovis* [17]. Additionally, the PCR of *M. bovis* Mbov_0732 gene (GenBank accession no.: CP002058) was performed to differentiate the attenuated strain P150 from P1, with this gene being deleted in P150 as previously reported [15].

The routine general bacterial culture for the nasal swabs, tissues and fluids from each calf was performed to detect other infectious agents like *P. multocida*, *M. haemolytica*, or *H. somni*. The samples were used to inoculate plates made with tryptic soy agar (with 5% newborn calf serum) and sheep blood agar plates. Colonies suspected to be the bacterial species of interest were further identified and sequenced by PCR with universe primers to 16S rRNA gene and sequencing [18].

The tissues from each calf were further tested for viruses associated with respiratory diseases like BoHV-1, BVDV, BRSV and BPI3 by using commercially antigen capture ELISA kits (#BIO K 340/5, Bio-X Diagnostics S.A.) for antigenic diagnosis according to the manufacturer’s instructions.

### 2.6. Gross Pathological Examination

Lesions on the surface or cross-sections of internal organs were examined post mortem. Subsequently, quantitative scoring was performed on the gross pathology of chest cavity, including changes in lung color, adhesion between the lung and chest wall, effusion of the pleura and pericardium, and lesions on the surface of trachea and lungs. The lung was further scored with a 35-point scoring system for evaluation of lesions in the seven lobes. The scoring was performed blindly by the same researcher according to a previously described method [15] to ensure scoring consistency and minimize observer bias.

### 2.7. Isolation and In Vitro Stimulation of Peripheral Blood Mononuclear Cells

PBMCs were isolated from blood samples of the infected calves using a Ficoll gradient (MP Biomedicals, Irvine, CA, USA), washed with phosphate-buffered saline, and suspended in RPMI-1640 complete medium with 10% fetal bovine serum at a volume equal to the original blood sample. Cell viability was determined by the trypan blue exclusion assay, and live cells were counted. Specifically, fresh PBMCs were seeded into a 12-well plate at a density of 2–4 × 10^6^ live cells per well. The PBMCs from the calves of P1 and P150 groups were re-stimulated in vitro with corresponding heat-killed (70 °C for 5 min) *M. bovis* strain at a dose of 5 μg protein per well, while PBMCs from mock-infected group were stimulated with equal volume PBS. After that, the cells were incubated at 37 °C in 5% CO_2_ for 72 h. Then, the cells were harvested and stored for microarray assay and identification with qPCR. In addition, whole blood sample was stimulated with heat-killed *M. bovis* strain at a dose of 5 μg per mL or PBS as descripted as above and cultured for 24 h at 37 °C in 5% CO_2_. Then, after a centrifugation at 1000× *g* for 5 min at 4 °C, the supernatant was collected and stored at –80 °C for analysis of IFN-γ and IL-17A production using commercial bovine IFN-γ ELISA kit (#EBC101g, NeoBioscience, Beijing, China) and bovine IL-17A ELISA kit (#VS0284B-002, Kingfisher, St. Paul, MN, USA), respectively, according to the manufacturers’ instructions.

### 2.8. Microarray Transcriptome Analysis of Peripheral Blood Mononuclear Cells

The stimulated individual PBMCs samples collected from all the nine calves at 7 dpi were evaluated by microarray analysis, with one microarray per calf. Briefly, PBMCs were centrifuged at 300× *g* for 5 min at 4 °C and lysed with 1 mL of TRIzol Reagent (Invitrogen, Carlsbad, CA, USA) by repeated pipetting. The total RNA was isolated by the method of Tizioto [19]. Subsequently, 1 μg of total RNA isolated from each calf sample was amplified with primers specific to the T7 promoter, labeled with cyanine 3 using the Quick Amp Labeling Kit (One-Color; Agilent, Santa Clara, CA, USA), and hybridized onto Agilent Bovine 4x44K Gene Expression Microarrays v2 (Agilent, Santa Clara, CA, USA). A total of 43,803 probes representing approximately 25,900 distinct bovine genes were arrayed. Raw signal intensities were normalized by the quantile normalization method with the GeneSpring GX v12.0 software, and low-intensity genes were filtered. The genes with flags detected in at least three out of nine samples were chosen for further analysis. The statistical analysis of the raw data of gene expression was performed by using R scripts (www.r-project.org) and Bioconductor packages (http://www.bioconductor.org) as described previously [20]. The genes with a fold-change higher than 2.0 and *p* value lower than 0.05 were considered as differentially expressed genes (DEGs) between the two groups. 

Hierarchical cluster analysis of DEGs was conducted using the software Cluster 3.0. The functional classification of Gene Ontology (GO) and Kyoto Encyclopedia of Genes and Genomes (KEGG) pathway analyses of DEGs were performed using the bioinformatics enrichment tools in the DAVID Bioinformatics Resources 6.8 (https://david.ncifcrf.gov/) [21]. The GO terms and KEGG pathways were considered significantly enriched when the *p* value was lower than 0.05.

Protein-protein interaction (PPI) network was constructed to identify the crucial genes that are actively involved in disease development [22]. In this study, the DEGs in P1 and P150 groups enriched in GO term analysis and KEGG pathway analysis were further submitted to Search Tool for the Retrieval of Interacting Genes website (STRING, http: //www.stringdb. org/) to analyze PPI. The PPI networks of these DEGs were visualized by also using the STRING database. In the PPI network, each node represents one gene, and lines between pairs of genes represent the relationship between the two corresponding proteins. The nodes having more connectivity with other genes are more vital in the PPI network. The top genes that are most frequently associated with other genes in each network were selected for further analysis.

### 2.9. Confirmation of Differentially Expressed Gene Transcription by qPCR

Nine DEGs in microarrays were selected for validation by qPCR, including three up-regulated DEGs in both P1 and P150 groups (*TRPV4*, *NOD1*, and *PIK3CB*), four up-regulated DEGs in the P1 group (*IL-17D, SYK, IL21R,* and *IL23R*), one down-regulated gene in the P1 group (*TLR4*), one up-regulated gene (*MDM2*) in the P150 group, and one non-modulated gene (*TLR2*). 

To further verify the expression changes of genes associated with Th17 differentiation induced by P1 infection, about 0.2 g of lung tissues from the experimental calves was ground immediately after adding some liquid nitrogen, then lysed with 1 mL of TRIzol Reagent (Invitrogen) by repeated pipetting. The total RNA was isolated by the method of Tizioto [19]. Primers specific to these genes were designed using the software Primer 5 (Table S1). Reverse transcription and qPCR reaction were performed by using the First Strand cDNA Synthesis Kit (#R223-01; Vazyme, Nanjing, China) and AceQ qPCR (SYBR Green Master Mix) kit (#Q111-02; Vazyme) according to the manufacturer’s instructions. The cDNA was synthesized with a 20 μL reaction system containing 50 pg/μL of total RNA, 1× HiScript II select qRt SuperMix II Mix, 0.5 μM Oligo(dT) 23VN, 2.5 ng/μL random hexamers and a corresponding volume of RNase free ddH_2_O. After mixing by pipetting, the reaction of reverse transcription was allowed to proceed at 50 °C for 15 min and at 85 °C for 10 s. The 10 μL qPCR reaction mixture consisted of 1.5 μL of cDNA template, 2.9 μL of RNase free water, 0.2 μL of upstream and downstream primers (the final concentration of 0.2 μM), 0.2 μL of 5× ROX_2_, and 5 μL of 2× AceQ qPCR SYBR Green Master Mix. All reactions were performed in duplicate using the standard ViiA 7 system (Applied Biosystems, Darmstadt, Germany) with a hold stage of UDG activation at 50 °C for 5 min and initial denaturation at 95 °C for 5 min, followed by an amplification step of 40 cycles of denaturation at 95 °C for 15 s, annealing at 60 °C for 30 s, and extension at 72 °C for 30 s (signal acquisition). The melting curve stage with a prescribed protocol (95 °C–60 °C–95 °C) was added after the amplification step. Delta Ct value (ΔCt = Ct _target genes_ − Ct_house keeping genes_) was estimated, followed by the calculation of ΔΔCt value (ΔCt _P1 or P150 group_ − ΔCt _mock-infected group_), and the relative expression was expressed as 2^(−ΔΔCt)^ [23].

### 2.10. Confirmation of Differentially Expressed Genes at Protein Level by Western Blot Assay

To further verify the result of P1-induced regulated genes at the protein level, about 0.2 g of lung tissues from the experimental calves was homogenized and lysed with RIPA lysis buffer (#P0013B, Beyotime Biotechnology, Shanghai, China) containing protease inhibitor and phosphorylation protease inhibitor. After centrifugation at 12,000× *g* for 5 min at 4 °C, the supernatant was collected and boiled (95 °C) for 10 min in SDS-PAGE Sample Loading Buffer (#P0015L, Beyotime Biotechnology). 

Equal amounts of proteins were separated by SDS/PAGE and transferred to a 0.2-μm PVDF blotting membrane (#10600023, GE Healthcare Life Science, Little Chalfont, Buckinghamshire, UK). After blocking with 5% BSA or 5% defatted milk in TBST buffer (10 mM Tris/HCl pH 7.4, 75 mM NaCl, 1 mM EDTA, 0.1% Tween-20), the membranes were probed with primary mouse antibody against STAT3 (#9139T, Cell Signaling Technology, MA, USA), and primary rabbit antibodies against phosphorylated STAT3 (pSTAT3, #9145S, Cell Signaling Technology), IL-6 (#abs135607), IL-17A (#abs124006) and IL-21R (#abs124325) (Absin Bioscience Inc., Shanghai, China), with mouse antibody against β-actin (#60008-1, Protech, Wuhan, China) as loading control. The secondary antibodies used were either anti-rabbit or mouse IgG depending on the primary antibodies. Immunoreactive bands were visualized by detection of chemiluminescence using Clarity Western ECL substrate (#1705060, Bio-Rad, Hercules, CA, USA) and the ChemiDocT XRS+ Molecular Imager (Bio-Rad), followed by analysis with the Image Lab software (Bio-Rad).

### 2.11. Statistical Analyses

Differences between the groups in the ordinal data, such as systematic and lung gross pathologic scores were analyzed by Mann-Whitney U test to compare two data sets or Kruskal-Wallis one-way analysis of variance (ANOVA) to compare more than two data sets. The statistical analyses were performed using GraphPad Prism version 7.0 for Windows (GraphPad Software, La Jolla, CA, USA). 

The correlation of crucial DEGs with the daily-recorded clinical traits as well as the systemic and lung gross pathologic scores was calculated by Pearson’s correlation (www.socscistatistics.com/tests/pearson/), and expressed by Pearson’s correlation coefficient r. The values of *p* < 0.05 were considered significantly different for Pearson’s correlation between a differentially expressed gene and its clinical traits; the higher the Pearson’s correlation coefficient (r), the stronger the correlation between them.

Results with *p* values less than 0.05 marked as *, less than 0.01 as **, and less than 0.001 as *** were considered as statistically significant differences.

### 2.12. Accession Number

The transcriptome data obtained from the microarray analysis have been submitted to the NCBI’s Gene Expression Omnibus (http://www.ncbi.nlm.nih.gov/geo/) with the GEO series accession number GSE110517.

## 3. Results

### 3.1. Occurrence of Clinical Signs in Infected Calves

On 3 dpi, the calves in the P1 group began to show clinical signs of different severity, which persisted for one week after infection, including lethargy, decreased appetite, increased nasal discharge, tachypnea, and cough. Then, they displayed lethargy, decreased appetite, continuous wasting and intermittent cough, and swollen joint (140#), while other signs gradually decreased and finally disappeared on 16 dpi (140#). Nevertheless, no apparent clinical abnormality was observed in other groups. 

**Figure 1:**
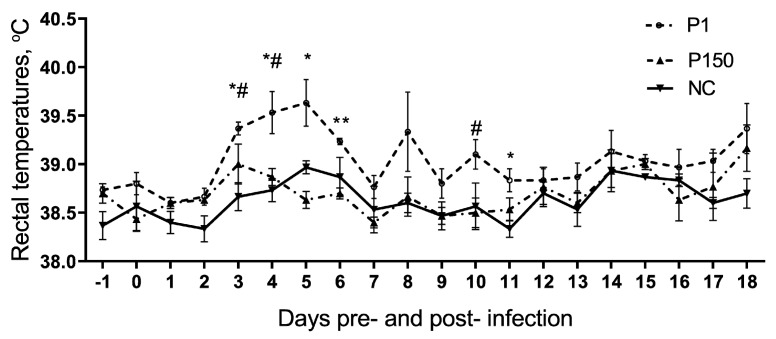
Rectal temperature curves of experimental calves pre- and post- infection by virulent and attenuated *Mycoplasma bovis* strains compared with mock-infected group (NC). The difference in the rectal temperature is statistically different between P1 and P150 (P150) groups (* *p* < 0.05 and ** *p* < 0.01) and between P1 and NC (# *p* < 0.05).

By comparing with the average background temperature before infection (–1 and 0 days pre-infection), the rectal temperature in the P1 group started to increase at 3 dpi and reached the peak at 5 dpi, with fluctuation between 0.6 °C–0.9 °C, followed by a decrease to the normal range (<0.5 °C) at 7 dpi, in contrast to the fluctuation < 0.5 °C in the other two groups. The comparison between groups was performed further, and the rectal temperatures in P1 group were significantly higher than P150 (0.4 °C–0.7 °C difference) and the mock-infected groups (NC group) (0.7 °C–0.8 °C difference) on 3 and 4 dpi (*p* < 0.05). On other days, P1 group was significantly higher than P150 on 5 (1 °C difference) and 11 dpi (0.3 °C difference) (*p* < 0.05) and 6 dpi (0.5 °C difference) (*p* < 0.01) and then NC group on 10 dpi (0.5 °C difference) (*p* < 0.05) (Figure 1).

**Figure 2:**
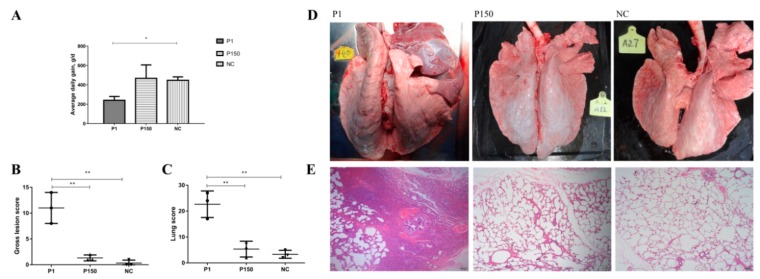
Average daily gain and pathological examination of calves of the three groups. (**A**) Average daily gain. (**B**) and (**C**) Evaluation of pathological severity with systemic gross score and lung score of the calves infected with either virulent *M. bovis* P1 (P1) or attenuated P150 strains (P150) with mock-infection control group (NC). (**D**) Gross lung lesion in the calves infected with *M. bovis* strains P1 and P150 compared with NC. (**E**) Histopathological images of lung tissues from the experimental calves stained by H&E. The lung tissues from the calves exhibited thickened alveolar septa, obliterated and collapsed (atelectatic) or dilated (emphysema) alveoli, shedding of bronchiolar epithelium and lymphohistiocytic infiltrates in P1 group, in contrast to intact architecture of alveoli and clear alveolar spaces in P150 and NC groups. Magnification = 40×.

The P1 group showed a significant decrease in average daily gain compared with the mock-infected group (*p* < 0.05), whereas the P150 group showed a similar average daily gain to the mock-infected group (*p* > 0.05) (Figure 2A). Additionally, the P1 group shed *M. bovis* nasally from 1 to 22 dpi, while the P150 group shed from 1 to 12 dpi. Meanwhile, the shedding ratio of 100% post-infection in nasal cavity lasted 1–15 days in the P1 group, but only 1–7 days in the P150 group (Table 1). The longer nasal shedding period of P1 than P150 in calves indicates the virulent strain had stronger ability of colonization and persistence in the host respiratory tract.

In the P1 group, the lung damage for three calves was primarily characterized with different degree of apparent consolidation, the right lobes were more severely affected than the left ones, and one of the calves had small purulent foci, similar to the early natural cases of *M. bovis* infection (Figure 2D and Figure S1). In contrast, the P150 and mock-infected groups exhibited no visible gross pathology (Figure 2D and Figure S1). The mean systemic score and lung lesion score were significantly higher in the P1 group than in P150 and mock-infected group (*p* < 0.05) (Figure 2B,C). The histopathological results were in agreement with those of gross pathological examination. The lung tissues in the P1 group showed significantly damaged alveolar architecture, thickened alveolar septa, obliterated and collapsed (atelectatic) or dilated (emphysema) alveoli, coupled with a high degree of lymphohistiocytic infiltrates, as well as exudates with inflammatory cells, degenerated epithelial cells, and intraluminal fibroblasts in bronchiolar (Figure 2E and Figure S2). However, the calves in the P150 and mock-infected groups maintained intact architecture of alveoli (Figure 2E, and Figure S2). All these different clinical and pathological traits verified the different degrees of virulence of P1 and P150 strains for calves. 

Meanwhile, *M. bovis* (P1 strain) was isolated from various samples of the P1 group, but P150 was only isolated from the tracheal mucus in the P150 group, with no *M. bovis* strains being isolated from the mock-infected group (Table 2). The results in the P1 group showed that the lung was the major site for virulent *M. bovis* colonization post infection. Besides, the IgG seroconversion was detected in the P1 and P150 group but not in the mock-infected group (Figure S3). The serology helped support the assertion that the infection of groups with P1 and P150 was successful, while the control group remained unexposed.

These samples failed to detect any other related co-infected or secondary pathogenic bacteria and viruses with routine bacterial culture isolation and viruses in the ELISA test (data not shown).

### 3.2. Differential Transcriptome Profiles

**Figure 3:**
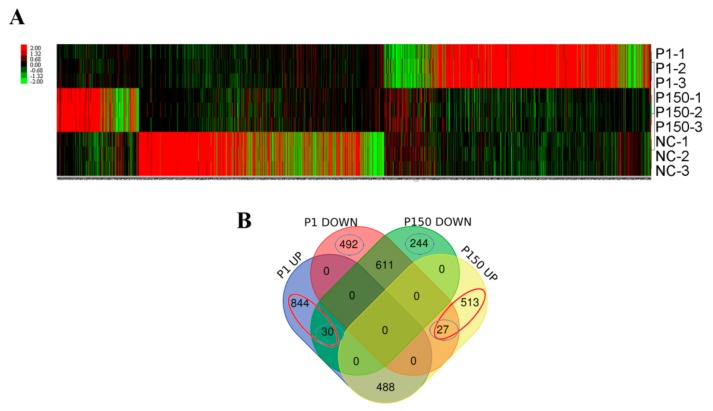
Profile of differentially expressed genes (DEGs) in the three groups. (**A**) Hierarchical heat map showing the expression dynamics of DEGs. P1-1 to 3 represent the calves of P1 group; P150-1 to 3, the calves of P150 group; NC-1 to 3, the calves of mock-infected group; (**B**) Venn diagrams of DEGs in peripheral blood mononuclear cells isolated from the calves of P1 and P150 groups versus mock-infected group. The red circle with “844” and “30” and the blue circles with “492” and “27” represent up- and down-regulated DEGs in P1 group after excluding common genes with similar trends of P150 group, respectively. Similarly, the red circle with “513” and “27” and the blue circles with ”44” and “30” represent up- and down-regulated DEGs in the P150 group after excluding common genes with similar trends of P1 group, respectively.

At 7 dpi, PBMCs from each calf were isolated and subjected to transcriptional microarray analysis. As a result, 22,277 probes representing 13,346 genes were chosen for further analysis. After normalization, DEGs were demonstrated in the hierarchical heat map by hierarchical cluster analysis (Figure 3A). Notably, there were more genes with modified expression in the P1 than P150 group. Furthermore, the two infected groups showed a different cluster distribution of DEGs from the mock-infected group, implying a correlation between DEGs and virulence. 

A quantitative comparison with the mock-infected group revealed the up-regulation of 1362 genes and down-regulation of 1130 genes in the P1 group in contrast to the up-regulation of 1028 and down-regulation of 885 genes in the P150 group. After excluding common genes with similar trends of P150 and P1 group, 874 and 519 up- and down-regulated DEGs were discovered in the P1 group as well as 540 and 274 up- and down-regulated DEGs in the P150 group, respectively (Figure 3B, Table S2). All these DEGs were used for further bioinformatic analyses to explore the pathogenic and immune mechanism related to the two *M. bovis* strains.

### 3.3. Bioinformatics Analysis of Differentially Expressed Genes of Peripheral Blood Mononuclear Cells in P1 Group

In the P1 group, 874 up-regulated DEGs versus mock-infected group were analyzed, and 279 of them were classified into 47 GO categories by the DVAID database, such as regulation of transcription from RNA polymerase II promoter, inflammatory response, regulation of apoptotic process, and so on. The top 15 genes included *IL-6, GSK3B, TP53, ARRB2, RAF1, STAT3, SYK, AMOT, BMP4, RBM5, RIPK2, HAX1, HCLS1, RAMP2,* and *SGK1* according to their enrichment in the GO categories (Table S3). Meanwhile, 118 genes of the 519 down-regulated genes were enriched in 29 GO categories, such as oxidation-reduction process, viral process, cell-cell adhesion, and so on (Table S3). The top 15 genes were *TLR4, TNF, UBC, RIPK1, INS, BIRC3, EIF4G1, CHRNA7, CRTC3, HSPA8, INSR, IRAK2, NFKBIA, TICAM2,* and *VDAC1*.

**Figure 4:**
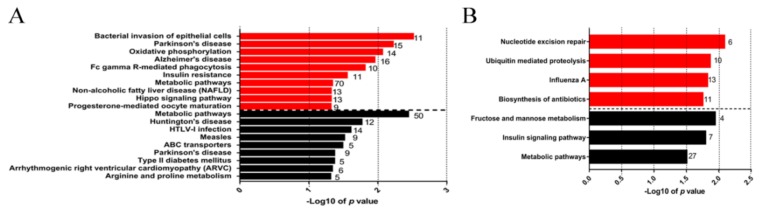
Kyoto Encyclopedia of Genes and Genomes pathways enriched with DEGs in peripheral blood mononuclear cells isolated from calves infected with different virulent *M. bovis*. (**A**) Pathways enriched with the up-regulated DEGs (red strips) and down-regulated DEGs (black strips) in P1 group versus mock-infected group; (**B**) Pathways enriched with the up-regulated DEGs (red strips) and down-regulated DEGs (black strips) in P150 group versus mock-infected group.

KEGG pathway analysis revealed that the P1 up-regulated DEGs vs mock-infected group were mostly enriched in bacterial invasion of epithelial cells, Fc gamma R-mediated phagocytosis, etc., with 35 genes being involved (Figure 4A). Meanwhile, the P1 down-regulated DEGs vs mock-infected group were mainly associated with ABC transporters, arginine and proline metabolism, with 10 genes being involved (Table S4).

**Figure 5:**
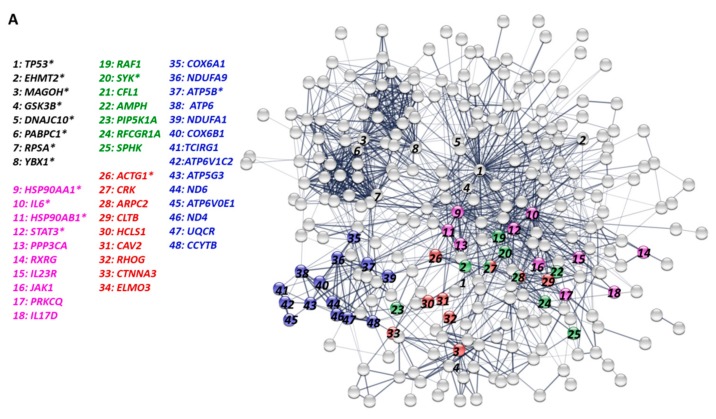

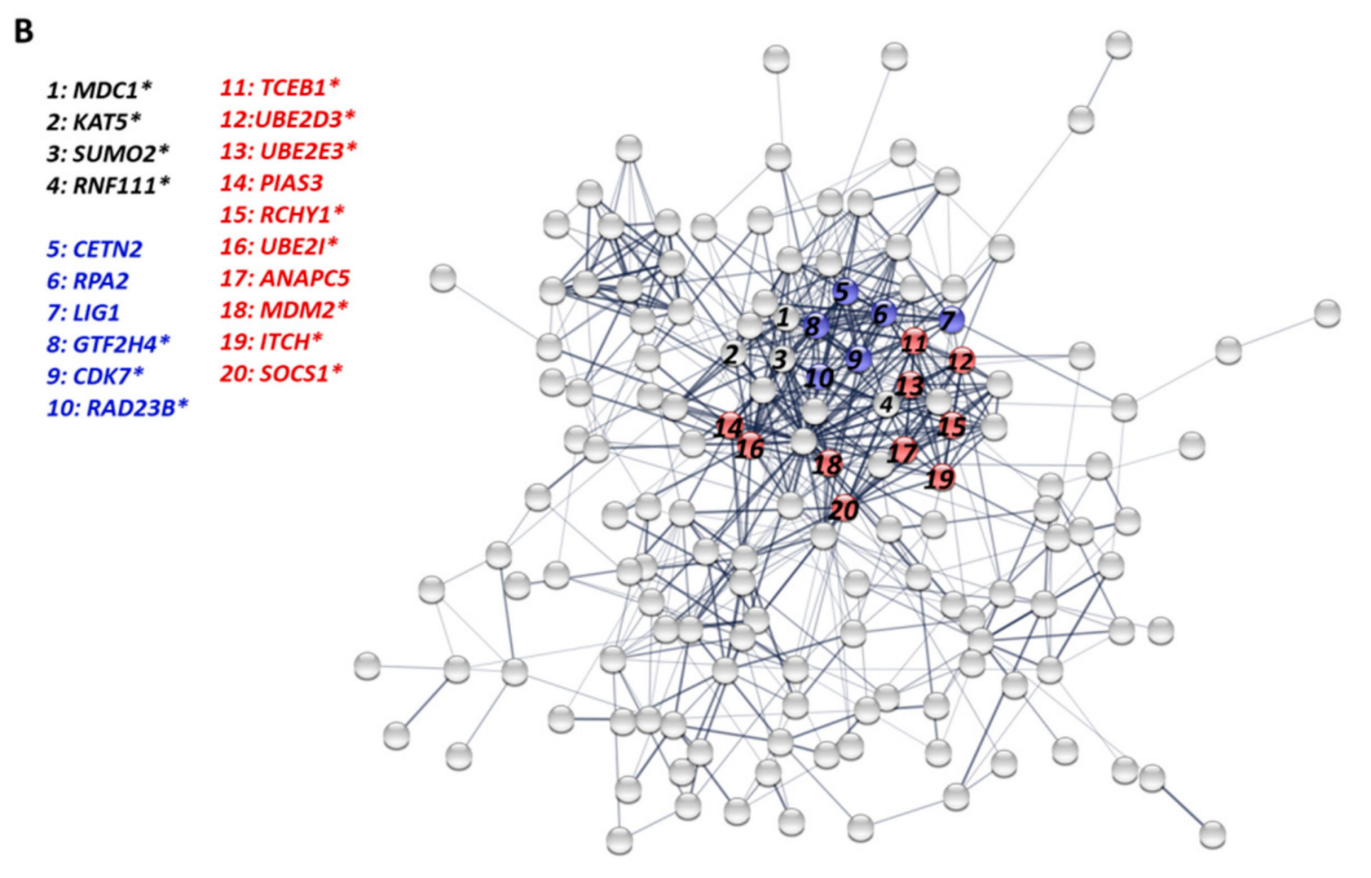
Protein-protein interaction (PPI) networks of DEGs in peripheral blood mononuclear cells isolated from infected calves. (**A**) The PPI network enriched with up-regulated DEGs in P1 group versus mock-infected group. The red circles and fonts represent the genes associated with the pathway of bacterial invasion of epithelial cells; green circles and fonts, Fc gamma R-mediated phagocytosis; blue circles and fonts, oxidative phosphorylation; pink circles and fonts, Th 17 cell differentiation pathway. * means the top 15 genes with the most connectivity in the PPI network. (**B**) The PPI network enriched with up-regulated DEGs in P150 group versus mock-infected group. The red circles and fonts represent the genes associated with the pathway of ubiquitin-mediated proteolysis; blue circles and fonts, genes associated with nucleotide excision repair; * means the top 15 genes with the most connectivity in the PPI network.

The 279 up-regulated DEGs of P1 vs mock-infected group enriched in GO term analysis and 35 genes in KEGG pathway analysis were further submitted to STRING (http: //www.stringdb.org/) website to analyze PPI. In the center of the PPI network were the genes involved in bacterial invasion of epithelial cells (red circles), Fc gamma R-mediated phagocytosis (green circles), and Th 17 cell differentiation (pink circles), with oxidative phosphorylation (blue circles) in another module of the PPI network (Figure 5A). The top 15 genes identified with the most connectivity in the PPI network were combined with the top 15 genes from GO terms and KEGG pathways, leading to the discovery of seven overlapping DEGs, including *SYK, TP53, IL-6, STAT3, ATP5B, GSK3B,* and *ACTG1*. The *SYK* gene appeared in all the three analyses, and the other genes were present in any two analyses. Four (*SYK, IL-6, STAT3,* and *GSK3B*) of the seven genes are associated with Th17 cell differentiation or IL-17 signaling pathway or both. 

Correspondingly, the top 15 genes with down-regulation in the P1 group and the most connectivity in the PPI network were extracted. After combining the three subsets of top 15 genes, nine overlapping hub DEGs were obtained, including *UBC, TNF, TLR4, INS, HSPA8, NFKBIA, INSR, NOS3,* and *BIRC3*, with the *UBC* gene in the center of the network (Figure S4).

### 3.4. Bioinformatics Analysis of Differentially Expressed Genes of Peripheral Blood Mononuclear Cells in P150 Group

Compared to mock-infected group, 13 up-regulated DEGs in the P150 group involved in the process of ribosome were excluded as the other processes may be “hidden” due to their strong interaction with each other. A total of 526 up-regulated DEGs in P150 vs mock-infected group were recognized by the DAVID database and 176 of them were classified into 33 GO categories, including signal transduction, positive regulation of GTPase activity, cell proliferation, and so on (Table S3). The relevant top 15 genes were *RPS27A, LIG1, LIG4, CCL2, JAK2, CDK7, CETN2, RAN, GTF2H4, MDM2, RPA2, RPL23A, SEH1L, TNFRSF1A*, and *UBE2I* according to their enrichment in the GO terms. 

These up-regulated DEGs in P150 vs mock-infected group were enriched in three pathways: nucleotide excision repair with six associated genes, ubiquitin-mediated proteolysis, and biosynthesis of antibiotics with 29 associated genes. 

The up-regulated DEGs in P150 vs mock-infected group, including 176 genes obtained from the GO analysis and 29 genes from the KEGG pathway analysis, were uploaded to the STRING website to generate the PPI network (Figure 5B). In the center of the PPI network were the genes involved in the pathways of ubiquitin-mediated proteolysis (red circles) and nucleotide excision repair (blue circles), implying that they might play an important role in the responses induced by P150. 

The combination of the top 15 genes in the PPI network, GO term and KEGG pathway analyses revealed 11 overlapping hub DEGs, including *UBE2I*, *GTF2H4*, *MDM2*, *RAD23B*, *CDK7*, *SOCS1*, *TCEB1*, *ITCH*, *UBE2D3*, *UBE2E3*, and *RCHY1*. Except for the association of *CDK7* and *GTF2H4* with nucleotide excision repair, the other nine genes are involved in the biological processes of ubiquitination and/or sumoylation. Furthermore, the four genes of *UBE2I*, *GTF2H4*, *MDM2*, and *CDK7* appeared in all the three analyses, indicating that they might be most critical in P150-induced response. Additionally, 258 down-regulated genes were identified, but only a few genes were enriched by analysis with DAVID database and STRING, and thus were not further analyzed.

### 3.5. Correlation of Differentially Expressed Genes with Clinical Signs

**Figure 6:**
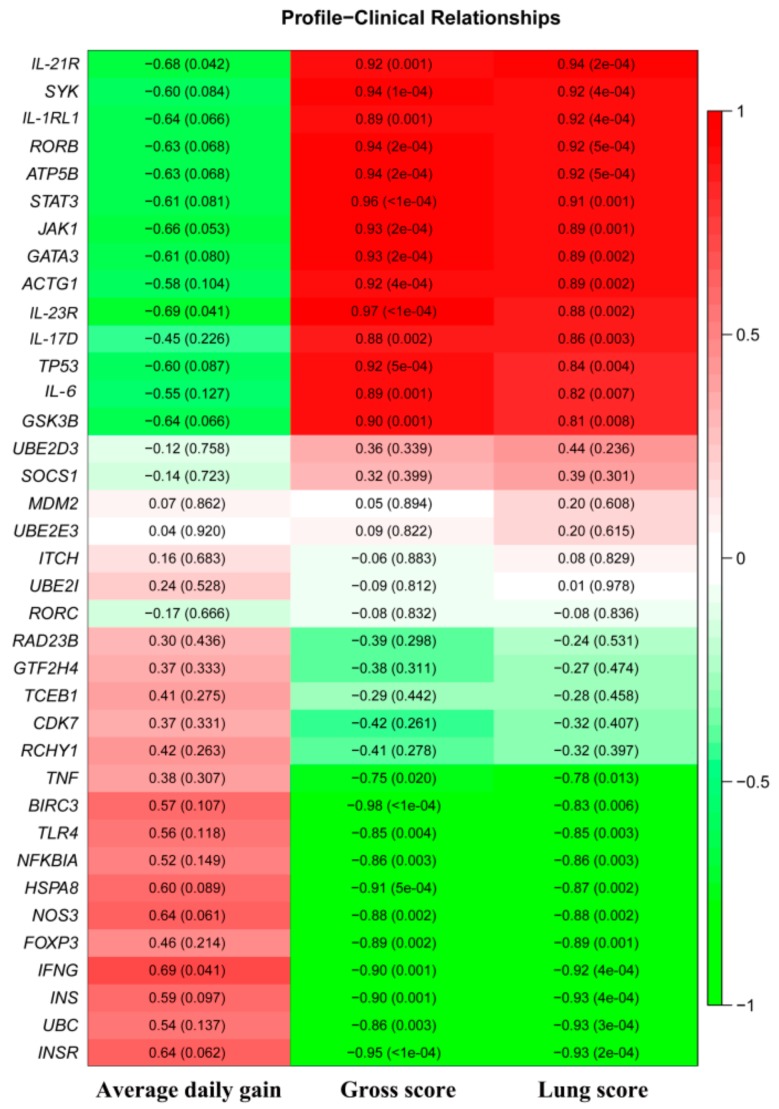
Correlation of DEGs with clinical trait scores. The color of each box represents the direction of correlation (red = positive correlation; green = negative correlation). The coefficient of r value is in the corresponding box, with *p* value in parentheses. Pearson’s correlation was used to determine the significance of correlation (*p* < 0.05) between the individual genes and clinical traits, including daily gain, gross score, and lung score.

The correlation analysis between DEGs and clinical traits (Figure 6) revealed a positive correlation of 14 DEGs (*ATP5B*, *JAK1*, *RORB*, *SYK*, *IL-17D*, *IL-23R*, *STAT3*, *GSK3B*, *IL-1RL1*, *ACTG1*, *TP53*, *IL-6*, *IL-21R*, and *GATA3*) with the general gross score and lung score of calves (*p* < 0.05). Meanwhile, 11 DEGs (*INS*, *INSR*, *UBC*, *TNF*, *NFKBIA*, *FOXP3*, *NOS3*, *HSPA8*, *TLR4*, *BIRC3*, and *IFNG*) were found to be negatively correlated with the general gross score and lung score of calves (*p* < 0.05). However, only *IFNG* gene exhibited a positive correlation with the daily gain of calves (*p* < 0.05). 

### 3.6. Validation of General Microarray Results and Genes Critical to Th17 Cell Differentiation in Lung Tissues

The qPCR was conducted to validate the transcription of nine genes selected from microarray analysis, and eight were confirmed by qPCR except for TLR4, which displayed no difference in the three groups, despite the microarray data suggesting down-regulation in the P1 group animals (Table S5). 

**Figure 7:**
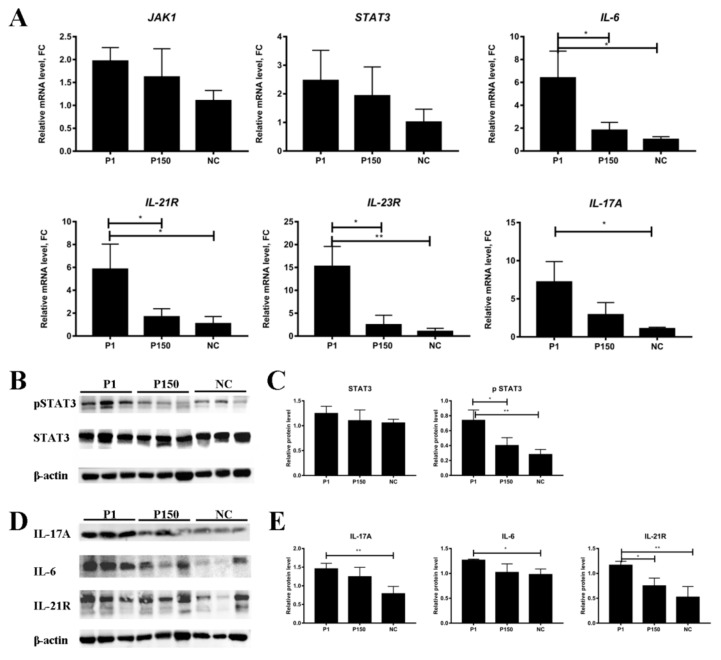
Verification of the expression of DEGs in lung tissues from the cattle infected with *M. bovis* by quantitative real-time polymerase chain reaction and Western blot. (**A**) Transcriptional level of genes involved in the pathway of Th17 cell differentiation. (**B**–**E**) Western blot assay detected expression level of proteins involved in the pathway of Th17 cell differentiation; β-actin represents the housekeeping gene. P1 represents as the P1 group, P150 as the P150 group, and NC as the mock-infected group, respectively.

Subsequently, six genes critical to Th17 cell differentiation pathway were tested by qPCR in the lung tissues from the three groups. Compared with the P150 or mock-infected group, P1 group showed a significant up-regulation of *IL-17A*, *IL-6*, *IL-21R*, and *IL-23R* (*p* < 0.05) in the lung tissues from the diseased cattle (Figure 7A), while no significant changes in the expression of *JAK1* and *STAT3* (*p* > 0.05).

The genes associated with the pathway of Th17 cell differentiation were further confirmed at the protein level (Figure 7B–E). Consistent with the results of qPCR and microarray analyses at the mRNA level, compared with the P150 or mock-infected group, the P1 group showed significantly increased expression of IL-21R and IL-6 (*p* < 0.05) at the protein level in lung tissues. Additionally, despite no significant change in the total expression (*p* > 0.05), the pSTAT3 displayed significantly higher expression in the P1 group than in the other two groups (*p* < 0.05). Furthermore, IL-17A, a dominant member of the IL-17 family, is closely associated with Th17 cell differentiation. Although it showed no significant expression difference in the PBMCs of calves at 7 dpi by microarray assay, Western blot analysis detected its enhancement in the lung tissues of P1 group at 60 dpi (Figure 7B).

### 3.7. Production of Blood IL-17A and IFN-γ in Experimental Calves 

**Figure 8:**
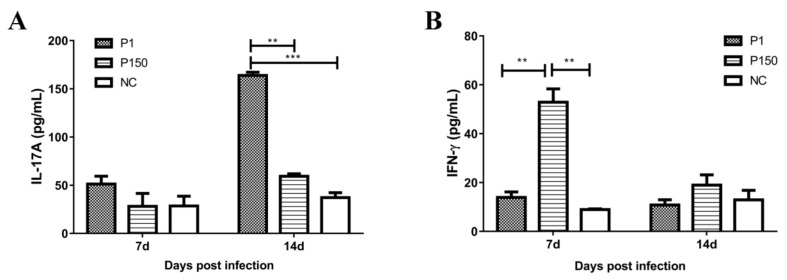
Blood IL-17A (**A**) and IFN-γ (**B**) concentrations in experimental calves. P1 represents as the P1 group, P150 as the P150 group, and NC as the mock-infected group, respectively.

To further confirm the results of lung tissues obtained with qPCR and Western blot and PBMCs analyzed with microarrays, IL-17A and IFN-γ were detected at 7 and 14 dpi in the whole blood of experimental calves. The three groups showed no significant difference of IL-17A at 7 dpi, which was consistent with the microarray assay results. However, when compared with the other two groups at 14 dpi, P1 group showed a significant increase in the blood IL-17A level (*p* < 0.05; Figure 8A), confirming the detection results in lung tissues by qPCR and Western blot analyses. Interestingly, the P150 group showed a significant increase over the P1 group in the blood IFN-γ level (*p* < 0.05) at 7 dpi, followed by restoration to the level of the mock-infected and P1 groups at 14 dpi (Figure 8B), whereas the fold change of *IFNG* was less than two times (fold change = 1.43, *p* < 0.05) in P150 versus P1 group in the microarray assay. 

## 4. Discussion

Despite previous demonstration of *M. bovis* P1 as a virulent strain and the derived *M. bovis* P150 as an attenuated and protective strain [15], the mechanisms of pathogenesis for P1 and immunity for P150 still remain unclear. Therefore, we conducted the in vivo experiment to reveal the underlying mechanism for offering novel insights into the pathogenic and immune mechanisms of the two strains. 

At 7 dpi, the calves generated apparent response to virulent *M. bovis.* The rectal temperature just completed the increase cycle (Figure 1), and other clinical sighs were different from that of P150 and mock-infected groups. Therefore, the differential transcriptional profile of bovine PBMCs at this time point would reveal the early response to infection. However, as described before [15], *M. bovis* pneumonia is a chronic disease. To observe typical lung lesions induced by *M. bovis* infection at less dose (10^9^ CFU/head) than before (10^10^ CFU/head), we observed the animals for 60 days after infection. Apart from clinical signs, the pathological observation at necropsy also indicated the significant difference in virulence of these two strains on calves—pneumonia with the features of lung consolidation, significantly damaged alveolar architecture, lymphohistiocytic infiltrates, and exudates in bronchiolar induced by P1, which is similar to the feature of moderate *M. bovis* pneumonia in natural infection, while in P150 group, similar to NC group, the intact alveolar and bronchial structures were observed. These indicated that the infection was successful which laid the foundation for the further study on interaction between DEGs and the clinical signs, or pathological changes of calves responding to *M. bovis* strains. 

### 4.1. Genes Most Likely Related to Mycoplasma bovis Pathogenesis in Calves

The bioinformatics analysis revealed that four (*SYK*, *IL-6*, *STAT3*, and *GSK3B*) in the seven hub up-regulated DEGs of P1 vs mock-infected group were involved in the pathways of Th17 cell differentiation or IL-17 signaling or both (Table S6). The correlation assay between pathological lesions and DEGs discovered 14 positively related genes, including the aforementioned seven genes and other seven genes: *IL-21R*, *IL-1RL1*, *RORB*, *JAK1*, *GATA3*, *IL-23R*, and *IL-17D*.

*SYK* (spleen tyrosine kinase) was among the top genes identified by the GO term, KEGG pathway and PPI network analyses. As a cytoplasmic tyrosine protein, SYK plays an important role in the pathophysiology of diseases associated with allergic inflammation and hyper-responsiveness, such as asthma [24] and arthritis [25], and has been predicted to be an attractive target for therapeutic kinase inhibitors. It regulates B cells by initiating the pathways of IP3 and diacyl glycerol via the translocation of activated transcript factors (cNFAT, NF-κB, and c-Jun/c-Fos) into the nuclei, thus modulating the expression of pro-inflammatory cytokines and promoting the growth and differentiation of T cells [26] SYK also couples with myeloid C-type lectin receptors to induce Th17 response in the presence of *IL-1*, *IL-6*, and *IL-23* [27] Therefore, the pathogenesis of *M. bovis*-induced diseases, such as pneumonia, might be associated with the overexpression of SYK-mediated reactivity in inflammatory and immune response.

The JAK1/STAT3 pathway plays a crucial role in the differentiation of Th17 cells. STAT3 is phosphorylated by JAKs, then translocated to the cell nuclei as a transcription activator. STAT3 regulates Th17 differentiation by directly binding to *IL-17A*, *IL-6*, and *IL-23R* [28,29]. In the present study, the overexpression of *JAK1*, *STAT3, IL-23R*, and *IL-17D* of PBMCs in mRNA level and increased pSTAT3 of lung tissue in protein level in P1 group indicated that JAK/STAT3 pathway was activated and might contribute to Th17 differentiation during *M. bovis* infection and related diseases. 

*IL-6*, derived from Th2 cells, drives the stimulation of B-cell and T-cell differentiation into Th2 cells [30] but induces pathological effect on chronic inflammation and autoimmunity when dysregulated continual synthesis. It is increased in the bronchoalveolar lavage fluid (BALF) and sera of patients with severe *M. pneumoniae* pneumonia [31,32]. Furthermore, *IL-6* is indispensable in triggering Th17 cell differentiation in fibroblasts, and *IL-17* produced by Th17 cells in turn induces *IL-6* production in target cells [33] and perform a pathogenic or protective function to the host depending on the atmosphere of cytokines. 

Glycogen synthase kinase 3 beta (GSK3B), a negative regulator of glucose homeostasis, has a potent therapeutic potential in the control of bacteria-induced inflammatory diseases [34,35,36,37]. The accumulation of GSK3B induces the production of *IL-6* by stimulating the PI3K/Akt pathway [38], and participates in the *IL-17* signaling pathway involved in the inhibition of autophagy [39] and pathological inflammation [40]. The GSK3B inhibitor reduces the production of *IL-6*, *TNFα*, *IL-17*, and *IL-1β* in the BALF of mice with lipopolysaccharide-induced acute respiratory distress syndrome, thus relieving the lung injury [41]. Therefore, the enhanced GSK3B expression in the P1 group suggested the association of GSK3B with the increased production of IL-6 and IL-17A as well as its involvement in the inflammatory lung injury of *M. bovis*-infected calves. 

ATP5B produces ATP from ADP in the presence of proton gradient across the membrane by electron transport complexes of the respiratory chain. Its overexpression in patients with asthma, coupled with the increased Th2-mediated response and decreased Th1-mediated response, promotes the cell proliferation and thickening of airway smooth muscle [42]. The overexpression of ATP5B in the P1 group (11.4-folds higher than P150 group) indicates that it might play an important role in respiratory disorders. Besides, *IL-1RL1* (alias ST2) could also induce Th17-mediated airway inflammation [43,44].

All the aforementioned up-regulated hub DEGs and other genes associated with Th17 cell differentiation of P1 vs. mock-infected groups suggest that Th17 response bias might be crucial for the development of *M. bovis*-related diseases. 

### 4.2. Pathogenic Th17 Biased Cell Response Is Induced by Virulent Mycoplasma bovis Strain in Calves

Th17 cells produce *IL-17*, a family of six members (*IL-17A* to *F*). A microenvironment with different cytokines and chemokines can lead to the Th17 differentiation into either protective Treg17 cells or pro-inflammatory pathogenic effector Th17 (Teff17) cells; the former is induced by *IL-6* and TGF-β, and suppresses the secretion of *IL-17A* and *IL-17F* [45,46]. Conversely, *IL-6*, *IL-23*, and *IL-1β* induce the differentiation of pathogenic Teff17 cells which contributes to the production of *IL-17A* and *IL-17F* [47]. Treg17 cells play an important role in maintaining mucosal barriers and pathogen clearance in the mucosal surfaces, whereas Teff17 cells are implicated to be involved in autoimmune and inflammatory disorders [28,47]. 

In this study, microarray assay at 7 dpi showed significant up-regulation of *IL-17D* in P1 vs. the mock-infected group, but no differential expression of *IL-17A* was confirmed by the blood test at the protein level representing *IL-17A* production status at an early stage of *M. bovis* infection. However, a significant difference was observed between them at 14 dpi in blood and 60 dpi in lung tissues in P1 group vs. mock-infected group, which is in accordance with the findings of pathogenic Th17 cell differentiation and therefore indicates a late response of this Th17 cell subset induced by the virulent P1 strain. Additionally, qPCR also confirmed the up-regulation of *IL-23R* and *IL-21R* in the PBMCs of the P1 group as well as *IL-6*, *IL-23R*, and *IL-21R* in the lung tissues of calves in the P1 group relative to the other two groups. Meanwhile, *IL-6* and *IL-21R* were confirmed to increase in the lung tissues by Western blot assay, which is also in agreement with the microarray results. Combined with the enhanced JAK1/STAT3, the increased expression of *IL-6, IL-17A*, *IL-17D, IL-23R,* and *IL-1RL1* probably contributed to the development of an atmosphere for the proliferation of pathogenic Teff17 cells. On the other hand, the down-regulation of *FOXP3* in the P1 group compared with the other groups confirmed the suppression of Treg17 response. Previous studies have shown that the repression of Treg17 cells may promote the over-responsiveness of pathogenic Teff17 cells [45,48]. Therefore, Teff17 over-responsiveness may have occurred in P1 group due to both the up-regulation of Teff17 cells and the down-regulation of Treg17 cells. However, it is unknown if the up-regulation of Teff17 cells would contribute to the pathogenesis or on the contrary, the lesion induced over-responsiveness of Teff17 cells. Some previous studies suggest the former possibility. As reported in systemic lupus, Treg17 deficiency in mice aggravated pulmonary vasculitis with increased Th17 cells [48]. Th17/Treg ratio was found to be higher in the patients with refractory than those with macrolide responsive *M. pneumoniae* pneumonia [49], and Tregs were reported to dampen the disease severity on mice induced by *M. pneumoniae* [50]. Despite two previous reports on the role of Th17/*IL-17* in *M. bovis* infection, they just performed a preliminary investigation and did not reach any conclusion about the association between Th17 subsets and the pathogenesis involved in the infection [8,13]. To our best knowledge, this is the first report concerning the correlation between pathogenic Th17 subsets and pathogenesis for *M. bovis* infection in cattle.

However, given the wide array of highly specialized and diverse T effector cell subpopulations, the involvement of lineage-specific pathogenic Th17 and Treg17 induced by *M. bovis* infection deserves to be further investigated in the future. 

### 4.3. Ubiquitination Might Be Critical to P150 Attenuation and Protective Effect

The Th1 response cytokine, IFN-γ, was verified at the protein level to be significantly higher in the blood samples from the P150 than P1 group, which was in agreement with the results of microarray analysis. In addition, a previous report discovered that after *M. pneumoniae* infection or immunization in mice, deficiency of IFN-γ resulted in Th2-type responses accompanied with immunopathologic reactions [51]. So, it might be a universe rule that IFN-γ production representing enhanced Th1 response might play a protective role against *Mycoplasma* infection, and therefore the strategy for development of novel vaccination against *Mycoplasma* species should prompt IFN-γ or Th1 responses [51]. 

Ubiquitination is an enzymatic post-translational modification where one ubiquitin molecule or a chain of ubiquitin molecules is attached to a substrate protein to further induce proteasomal degradation. This study found eight hub up-regulated DEGs in the P150 group, including five E3 ubiquitin ligases (E3s), *ITCH*, *MDM2*, *RCHY1*, *TCEB1*, and *SOCS1,* and three ubiquitin-conjugating enzymes (E2s), *UBE2D3*, *UBE2I*, and *UBE2E3*. ITCH inhibits Th2 differentiation by inducing ubiquitination of JunB and c-Jun, which are transcription factors involved in Th2 differentiation and the development of Th2 cytokines [52]. It also negatively regulates IL-17-mediated colonic inflammation and carcinogenesis by inducing RORC ubiquitination [53], a key transcription factor in the differentiation of Th17 cells [47]. This result was consistent with a depressed expression of *RORC* in P150 vs. mock-infected group in the present study (Table S6). Therefore, the overexpression of ITCH might be involved in suppression of Th2 and Th17 cell differentiation and promotion of Th1 response by mediating the ubiquitination of RORC in P150 group. MDM2 (P53 E3 ubiquitin protein ligase homolog) also inhibits the differentiation of CD4 naïve cells to Th2 cells by driving GATA3 poly ubiquitination [54,55]. SOCS1 was reported to maintain FOXP3 expression in Treg cell integrity and suppressing *IL-17* production through STAT3 [56]. UBE2D3 (ubiquitin-conjugating enzyme E2 D3) exerts the effect of immune modulation such as antiviral infection by promoting the covalent conjugation of polyubiquitin chains to RIG-I and production of IFN-α and IFN-β [57]. Besides, RAD23B, involving in ubiquitin-activation, was also up-regulated in the P150 group (Table S6). However, the interaction between these ubiquitin ligases and their target proteins needs to be further studied in the future. 

Collectively, the enhanced expression of enzymes associated with ubiquitination process and production of IFN-γ in P150 group indicated that there might be some interactions of the Th1 cytokine secretion with ubiquitination, which might contribute to the restraint of Th17 and Th2 responses in the host post-infection.

### 4.4. The Relevance of the Experimental Infection to the Natural Disease 

There are mainly two differences between experimental *M. bovis* infection and natural pneumonia. First, the clinical signs such as fever occurred earlier in experimental *M. bovis* infection than in natural diseases, which are believed to be chronic. In natural disease cases, it usually takes a two-week-incubation time for *M. bovis* to replicate to the number sufficient to cause the disease, while in the experimental infection, a large enough dose is adopted to cause the disease and thereby only a short incubation time of 3–5 days is usually needed. In the present study, the onset of fever occurred at 3 dpi and resolved at 7 dpi. This is in agreement with the results reported by other publications that the median time to resolve the high temperature is eight days post challenge [58]. Second, the natural *M. bovis* pneumonia was not only characterized as extensive consolidation but also foci of caseous necrosis. Except for cattle, there is no animal model that can replicate *M. bovis* pneumonia successfully. However, experimental infection cases in cattle usually replicated the pathology of extensive consolidation but not extensive foci of caseous necrosis [3,59,60,61,62,63], despite sometimes accompanied with multiple necrotic foci [60], abscesses, and necrosis [59,61] in some infected calves. These reports are consistent with our observations in most calves of P1 group. Co-infection of *M. bovis* with BoHV-1 could aggravate multifocal white nodules containing caseous material [64]. The general pathogenic model for *M. bovis* pneumonia is that *M. bovis* causes the primary damage in respiratory tract and then the secondary pathogens, such as *P. multocida*, and *M. haemolytica*, synergistically result in the final severe outcome of this disease. This was partially supported by an early study demonstrating that the most severe disease and highest degree of pneumonic consolidation was induced first by intranasal inoculation of *M. bovis* and one day later by inoculation of *P. haemolytica* [65]. However, the exact mechanism is not clear yet. Since lung consolidation is the early and basic pathology of *M. bovis* infection, we believe that there is a high relevance between our experimental infection and natural disease. 

Meanwhile, this research has limitations in explanation of difference in cytokine expression. Both PBMCs and lung tissues are composed of multiple cell types. It is not clear whether it is the number difference of cell populations or the expression difference of genes on a per-cell basis induced by the infection of P1 or P150 that contributes to the difference in IL-17A or IFN-γ levels. However, this “-omics” study describes the whole situation and the final outcome of change in these cytokine levels, which would be of significance for understanding the induced immunity or pathogenesis of *M. bovis* infection. 

## 5. Conclusions

In this study, a comprehensive analysis was performed on transcriptome data and its association with clinical signs, pathological changes as well as blood cytokines in calves infected with virulent and attenuated *M. bovis* strains. The integrated data demonstrated that pathogenic Teff17 response was enhanced and Treg17 response was suppressed by infection of the virulent *M. bovis*, whereas the attenuated P150-induced immunity might be associated with the stimulation of Th1 response. This research improves the understanding of *M. bovis*-induced pathogenesis and immunity that could be used in the development of control measures against *M. bovis* associated diseases. 

## Figures and Tables

**Table 1:** Number of nasal swabs from experimental calves positive for *Mycoplasma bovis* culture.

Groups	Days Post Infection								
	0	1	3	5	7	12	15	18	22
P1	0/3	3/3	3/3	3/3	3/3	3/3	3/3	2/3	2/3
P150	0/3	3/3	3/3	3/3	3/3	1/3	0/3	0/3	0/3
NC	0/3	0/3	0/3	0/3	0/3	0/3	0/3	0/3	0/3

**Table 2:** Isolation of *M. bovis* from the samples of experimental calves at the end of observation period (60 days post-infection).

Groups	Animal No.	Tracheal Mucus	Lung	Lung Lymph Nodes	Pleural Fluid	Joint Fluid
P1	140#	-	P1	-	-	P1
	98#	-	P1	P1	-	-
	65#	-	P1	-	-	-
P150	142#	-	-	-	-	-
	A20#	P150	-	-	-	-
	A12#	-	-	-	-	-
Mock-infection	91#	-	-	-	-	-
	173#	-	-	-	-	-
	A27#	-	-	-	-	-

## Supplementary Materials

The following are available online at https://www.mdpi.com/2073-4425/10/9/656/s1. Figure S1: Grossly visible lung lesions of all nine calves infected with the virulent and attenuated *Mycoplasma bovis* strains and mock-infected with PPLO medium, Figure S2: Histopathologic images of lung tissues from all calves infected with the virulent and attenuated *Mycoplasma bovis* strains and mock-infected with PPLO medium. Thickened alveolar septa, lymphohistiocytic infiltrates (A to C), obliterated and collapsed (atelectatic) or dilated (emphysema) alveoli (A and B), shedding of bronchiolar epithelium (A and C), exudate in bronchiolar containing different degree of inflammatory cells, degenerated epithelial cells and intraluminal fibroblasts (A to C) were visible in the lungs of P1 group. Intact architecture of alveoli in P150 (D to F) and mock-infected group (G to I) were observed. Magnification = 40 x or 100 x (panels in the upper right corner), Figure S3: The serum IgG levels of calves infected with the virulent and attenuated *Mycoplasma bovis* strains and mock-infected with PPLO medium. * *p* < 0.05, ** *p* < 0.01 and *** *p* < 0.001 for the comparison of serum IgG of P1 group with mock-infected group, while # *p* < 0.05, ## *p* < 0.01 and ### *p* < 0.001 for the comparison of P1 group with mock-infected group, Figure S4: Protein-protein interaction (PPI) networks of down-regulated differentially expressed genes in the peripheral blood mononuclear cells isolated in P1 (A) and P150 (B) group. (A) The red circles and fonts represent the genes associated with the pathway ABC transporters, green with NF-κB signaling pathway, and blue with arginine and proline metabolism. (B) The red circles and fonts represent the genes associated with the pathway fructose and mannose metabolism, and blue with oxidative phosphorylation. * means the top genes with the most connectivity in the PPI network, Table S1: Sequence of oligonucleotide primers used for quantitative polymerase chain reaction in the present study, Table S2: Differentially expressed genes in the peripheral blood mononuclear cells isolated from the calves of P1 and P150 groups compared with mock-infected group, Table S3: Gene Ontology terms enriched in the differentially expressed genes in the peripheral blood mononuclear cells isolated from the calves of P1 and P150 groups versus mock-infected group, Table S4: Kyoto Encyclopedia of Genes and Genomes pathways enriched in the differentially expressed genes in peripheral blood mononuclear cells isolated from the calves of P1 and P150 groups versus mock-infected group, Table S5: Verification of the expression of differentially expressed genes in the peripheral blood mononuclear cells isolated from the calves infected with different virulent strains by quantitative real-time polymerase chain reaction compared with mock-infected group, Table S6: Details of the hub and related differentially expressed genes in the peripheral blood mononuclear cells of the P1 group and P150 group versus mock-infected group, respectively, Table S7: Abbreviations.
