# Supplementary material for: Calves Infected with Virulent and Attenuated Mycoplasma bovis Strains Have Upregulated Th17 Inflammatory and Th1 Protective Responses, Respectively
Source: Genes (Basel). 2019 Aug 28;10(9):656. doi: 10.3390/genes10090656 (PMC6770603; doi:10.3390/genes10090656)
Supplement: Supplementary file 1 [file genes-10-00656-s001.zip › supplement/Table S1.docx]

| Gene name | Accession number | Forward primer sequence | Reverse primer sequence | Amplicon size |
| --- | --- | --- | --- | --- |
| 18SrRNA | AF176811 | CGGGGAGGTAGTGACGAAA | CCGCTCCCAAGATCCAACTA | 195 |
| GAPDH | XM_019960295.1 | ACCCAGAAGACTGTGGATGG | CAACAGACACGTTGGGAGTG | 178 |
| ACTB | NM_173979.3 | GACATCCGCAAGGACCTCTA | ACATCTGCTGGAAGGTGGAC | 205 |
| TRPV4 | NM_001192385 | TGCGGGACAAGTGGCGGAAGT | CGGTAAGGGTATGGCGGAGTG | 133 |
| NOD1 | NM_001256563 | CATCTGGTCACTCACATCCGAAAC | ACACCTCCTCGCCCTTGCTC | 163 |
| PIK3CB | NM_001206047 | TTGCAAGTCAGTGGGAGAGTAG | GTGGTAAAGGAAGAGGAAGGTT | 208 |
| SYK | NM_001037465 | CCTCCACAAAGTAAACCCGTCCC | ATGCTTGGTGTTCAAATGCAACC | 187 |
| IL17D | XM_002691866 | AGTGAGAGAAAGGCACACCC | CAGCCCTCCCATGACCTAAC | 216 |
| IL21R | NM_001193179 | CGGTTTACAGCCAGGAGAAG | CCAGGTTTAGGGCAGGGTAG | 132 |
| IL23R | NM_001127172 | CAAACTCTGCTGCCTGATGA | CGATTGAAGTGGCTTTCCA | 110 |
| MDM2 | NM_001099107 | TGACCGAGGTCCTGCCGTTTT | CCCCGAGGCTCCCCAGTTTCC | 163 |
| TLR4 | NM_174198 | TGCTGGCTGCAAAAAGTATG | TTACGGCTTTTGTGGAAACC | 213 |
| IL6 | NM_173923 | TCCTTGCTGCTTTCACACTC | CACCCCAGGCAGACTACTTC | 129 |
| IL17A | NM_001008412 | TGGTGGCTCTTGTGAAGGCAGG | TCAGGGTCCTCATTGCGGTGGA | 193^[[1]](#footnote-1)^ |
| JAK1 | NM_001206534 | CCAGCTCGCTGGAGTATCTG | CGCATCCTGGTGAGAAGGT | 262 |
| STAT3 | NM_001012671 | TTCTTCCTTTTCCCATCGACC | TTATCACTAAGCCTTTGCCCC | 271 |

1. GondairaS, Higuchi H, Iwano H, Nakajima K, Kawai K, Hashiguchi S, Konnai S, Nagahata H. Cytokine mRNA profiling and the proliferative response of bovine peripheral blood mononuclear cells to Mycoplasma bovis. 2015. Vet Immuno Immunop 165:45-53 [↑](#footnote-ref-1)
