# Supplementary material for: Calves Infected with Virulent and Attenuated Mycoplasma bovis Strains Have Upregulated Th17 Inflammatory and Th1 Protective Responses, Respectively
Source: Genes (Basel). 2019 Aug 28;10(9):656. doi: 10.3390/genes10090656 (PMC6770603; doi:10.3390/genes10090656)
Supplement: Supplementary file 1 [file genes-10-00656-s001.zip › supplement/Table S3.docx]

| **DEGs** | **GO Term enriched** | **Count** | ***p* Value** | **Genes** |
| --- | --- | --- | --- | --- |
| **P1 up-regulated** | GO:0045944~positive regulation of transcription from RNA polymerase II promoter | 55 | 0.022 | LMO4, FOXO4, YBX1, KDM1A, OSR2, GALR2, HSF4, SOX17, PHOX2B, SSBP4, MYO6, YY1, TP53, DMRT1, SIX5, IFI16, HLTF, HHEX, NCOA1, HOXC11, SPDEF, RIPK2, ZNF382, PEG3, HAX1, SOX1, CREM, ABLIM3, NR3C1, POMC, TTC5, NR2C2, ATF2, NFAT5, PPP3CA, NR1H3, BMP4, ASXL2, IL6, SUB1, HCLS1, RAF1, TEAD2, WWTR1, STAT3, PLAC8, HOXB4, PKNOX1, ATF3, CSRNP3, MEOX1, GSK3B, RBM14, PARP1, TCF12 |
|  | GO:0000122~negative regulation of transcription from RNA polymerase II promoter | 44 | 0.011 | WWC2, PRDM16, CALR, YBX1, ATF2, ZNF174, KDM1A, OSR2, TCERG1, CGGBP1, DDX20, HSF4, E4F1, SOX17, TPR, NR2F2, TCF25, DNAJA3, NR1H3, MDFI, BMP4, TXNIP, SATB1, ZNF280D, YY1, HCLS1, TP53, DMRT1, PHF12, IFI16, WWTR1, EHMT2, STAT3, SUZ12, HHEX, HOXB4, ATF3, CD36, DLL4, SPDEF, CUX2, PARP1, ZNF382, PEG3 |
|  | GO:0006954~inflammatory response | 24 | 0.046 | F11R, IL6, RARRES2, PLP1, IL23R, TLR1, AXL, TLR5, IFI16, TLR6, HNRNPA0, EPHA2, S100A12, PLAA, APOL3, S1PR3, IL17D, PRKCQ, GGT5, CYBB, CXCL13, RIPK2, MGLL, SYK |
|  | GO:0043065~positive regulation of apoptotic process | 21 | 0.026 | BMP4, TXNIP, ING5, FGD1, IL6, ING2, TP53, CTLA4, ARHGEF16, RBM5, NCOA1, CSRNP3, MAP3K9, BNIP3L, SPDEF, RIPK2, DDX20, CLIP3, IGFBP3, DNAJA3, FGD3 |
|  | GO:0042981~regulation of apoptotic process | 19 | 0.004 | GDF3, BMP3, SGK1, BCL2L14, HAX1, TP53, SKP2, RBM5, RAF1, MALT1, CALR, CDK5, MAGED1, PRELID1, BNIP3L, ROBO4, PPP1R13B, IGFBP3, RBM25 |
|  | GO:0010628~positive regulation of gene expression | 18 | 0.048 | SRPK2, RAMP2, PLP1, IL6, TP53, CALR, RIMS1, STAT3, RBM4B, APOB, OSR2, ARRB2, DLL4, UBAP2L, CUX2, EIF2AK3, FN1, MT3 |
|  | GO:0006413~translational initiation | 16 | 6E-04 | RPSA, RPL35A, EIF1AD, PAIP1, RPL27A, EIF2A, RPL38, RPL29, RPS28, RPL32, DHX29, EIF1AX, RPL3L, EIF3I, RPL37A, PABPC1 |
|  | GO:0006974~cellular response to DNA damage stimulus | 16 | 0.028 | SGK1, TAOK1, YY1, TP53, CTLA4, TOPBP1, MACROD1, HERC2, RAD50, ATF2, SPDYA, RASSF1, USP10, NEK4, PARP1, UBE2T |
|  | GO:0006364~rRNA processing | 16 | 0.034 | RPL35A, RPSA, TSR1, RPL27A, RPL38, RPP14, RPL29, EBNA1BP2, WDR75, WDR18, RPS28, RPL32, RPL3L, DDX21, RPL37A, XRN2 |
|  | GO:0000398~mRNA splicing, via spliceosome | 16 | 0.045 | DHX9, CSTF3, CRNKL1, TRA2B, MAGOH, SNRPB2, RBM5, HNRNPA0, YBX1, HNRNPL, SRRT, SNRNP200, USP49, SNRNP35, PABPC1, DHX32 |
|  | GO:0001525~angiogenesis | 16 | 0.047 | SRPK2, RAMP2, PDGFA, ATP5B, LEPR, NRXN1, ARHGAP24, PKNOX1, LAMA5, DLL4, ROBO4, SOX17, ANGPT2, EIF2AK3, FN1, SYK |
|  | GO:0000184~nuclear-transcribed mRNA catabolic process, nonsense-mediated decay | 14 | 0.001 | RPL35A, RPSA, UPF2, MAGOH, RPL27A, SMG1, RPL38, CTIF, RPL29, RPS28, RPL32, RPL3L, RPL37A, PABPC1 |
|  | GO:0001666~response to hypoxia | 14 | 0.028 | RAMP2, ND4, CYTB, RAF1, POSTN, TRH, DDIT4, ALAS2, CA9, PKLR, NOS2, ANGPT2, MT3, MB |
|  | GO:0051056~regulation of small GTPase mediated signal transduction | 13 | 0.01 | RHOJ, ARHGDIG, FGD1, GDI2, ARHGEF16, ARHGAP24, STARD8, CHN2, RHOT2, AMOT, RHOG, FGD3, ARHGDIB |
|  | GO:0006396~RNA processing | 11 | 0.007 | HNRNPL, DHX9, CSTF3, CRNKL1, DHX29, PABPC4, U2SURP, RBM5, DDX20, XRN2, RTCA |
|  | GO:0030334~regulation of cell migration | 10 | 0.004 | PARD6B, SGK1, PLXNA3, LAMA5, LMO4, ROBO4, MINK1, AMOT, CDK5, FLNA |
|  | GO:0030168~platelet activation | 10 | 0.05 | PRKCQ, IL6, ARRB2, AXL, COL1A2, RAF1, PIK3R5, FLNA, RHOG, SYK |
|  | GO:0050731~positive regulation of peptidyl-tyrosine phosphorylation | 9 | 0.021 | IL6, CD36, CCK, HAX1, ARRB2, HCLS1, TP53, RIPK2, SYK |
|  | GO:0001570~vasculogenesis | 8 | 0.008 | RAMP2, HHEX, CCM2, AMOT, HEG1, TEAD2, SOX17, EPHA2 |
|  | GO:0033138~positive regulation of peptidyl-serine phosphorylation | 8 | 0.026 | IL6, HAX1, ARRB2, HCLS1, GSK3B, RIPK2, RAF1, AKAP9 |
|  | GO:1900034~regulation of cellular response to heat | 8 | 0.036 | HSP90AB1, RPA1, HSP90AA1, RAE1, GSK3B, DNAJC7, TPR, NUP43 |
|  | GO:0016236~macroautophagy | 8 | 0.039 | TCIRG1, MFN1, ULK1, NBR1, TOMM20, TOMM40, ATG3, GABARAP |
|  | GO:0045454~cell redox homeostasis | 8 | 0.041 | CYBB, IL6, PTGES2, TXNDC15, DNAJC10, NOS2, GLRX2, GLRX |
|  | GO:0043401~steroid hormone mediated signaling pathway | 7 | 0.031 | BMP4, RXRG, RORB, HNF4G, NR2F2, NR2C2, NR1H3 |
|  | GO:0006606~protein import into nucleus | 7 | 0.033 | TXNIP, RAE1, CFL1, PPP3CA, TPR, ING1, STAT3 |
|  | GO:0001933~negative regulation of protein phosphorylation | 7 | 0.041 | CCNB1, INPP5K, ARRB2, DNAJC10, CTDSP2, WWTR1, IGFBP3 |
|  | GO:0032092~positive regulation of protein binding | 7 | 0.041 | HSP90AB1, BMP4, GSK3B, RFNG, RAPGEF2, CDK5, ADD1 |
|  | GO:0005977~glycogen metabolic process | 6 | 0.006 | PPP1CA, PPP1R2, PYGM, GSK3B, SLC37A4, PPP1CC |
|  | GO:0000381~regulation of alternative mRNA splicing, via spliceosome | 6 | 0.02 | HNRNPL, MAGOH, TRA2B, RBM5, WTAP, RBM25 |
|  | GO:0030522~intracellular receptor signaling pathway | 6 | 0.02 | NCOA1, RORB, HNF4G, NR2F2, STAT3, NR1H3 |
|  | GO:0071310~cellular response to organic substance | 5 | 0.005 | SYP, PSAP, CUX2, CALR, CALB1 |
|  | GO:0001578~microtubule bundle formation | 5 | 0.019 | PRC1, PSRC1, MAP2, CDK5RAP2, ZNF207 |
|  | GO:0007616~long-term memory | 5 | 0.028 | SGK1, EHMT2, CALB1, RGS14, BTBD9 |
|  | GO:0090004~positive regulation of establishment of protein localization to plasma membrane | 5 | 0.035 | ARHGEF16, CLIP3, NRXN1, RHOG, EPHA2 |
|  | GO:0015991~ATP hydrolysis coupled proton transport | 5 | 0.043 | TCIRG1, ATP6V1C2, ATP6V0E1, ATP5B, ATP5G3 |
|  | GO:0002755~MyD88-dependent toll-like receptor signaling pathway | 5 | 0.047 | CD36, TLR1, TLR5, TLR6, TAB3 |
|  | GO:0045793~positive regulation of cell size | 4 | 0.007 | HSP90AB1, KDM1A, HSP90AA1, AMOT |
|  | GO:0007212~dopamine receptor signaling pathway | 4 | 0.015 | ARRB2, GSK3B, ADCY6, NSG1 |
|  | GO:0016571~histone methylation | 4 | 0.032 | SUZ12, SATB1, PRMT7, EHMT2 |
|  | GO:0031333~negative regulation of protein complex assembly | 4 | 0.032 | ULK1, LMO4, GSK3B, RAF1 |
|  | GO:0050870~positive regulation of T cell activation | 4 | 0.037 | CD47, PRKCQ, CCDC88B, MALT1 |
|  | GO:0007043~cell-cell junction assembly | 4 | 0.037 | PARD6B, TLN2, AMOT, HEG1 |
|  | GO:0019370~leukotriene biosynthetic process | 4 | 0.048 | GGT5, PRG3, LTA4H, SYK |
|  | GO:0033210~leptin-mediated signaling pathway | 3 | 0.023 | LEPR, STAT3, MT3 |
|  | GO:0006013~mannose metabolic process | 3 | 0.032 | MAN2A1, MAN2B1, PMM2 |
|  | GO:0001945~lymph vessel development | 3 | 0.041 | HEG1, NR2F2, SYK |
|  | GO:0046903~secretion | 3 | 0.041 | PTGES2, CA9, RIMS1 |
| **P1 down-regulated** | GO:0055114~oxidation-reduction process | 26 | 0.008 | CYP2U1, TH, EGLN3, HR, P4HA2, P4HA1, P4HA3, NOS3, HHIP, DUS3L, PDPR, AIFM2, MICAL3, IDO2, COX4I2, SOD3, DHRS7, NNT, CYP26C1, TXNDC8, PRDX6, TXNRD3, KDM8, OXNAD1, CYP4F3, GLYR1 |
|  | GO:0016032~viral process | 15 | 0.019 | CRTC3, ABCE1, NUP133, NUP160, HCK, NFKBIA, VDAC1, RTN3, EIF4G1, PSMB4, GBF1, SND1, NPM1, TCEB1, HSPA8 |
|  | GO:0098609~cell-cell adhesion | 14 | 0.02 | ALDOA, ZC3HAV1, VAPB, BAIAP2, EPS15L1, EIF4G1, CORO1B, STXBP6, PRDX6, SND1, TJP2, HSPA8, NUDC, CEACAM1 |
|  | GO:0000086~G2/M transition of mitotic cell cycle | 10 | 0.008 | CCNB1, NES, KDM8, UBC, CETN2, TPD52L1, DCTN1, WEE1, TUBB4B, PPP2R2A |
|  | GO:0007249~I-kappaB kinase/NF-kappaB signaling | 9 | 1E-04 | IRAK2, TNF, RIPK1, TICAM2, UBC, TLR4, BIRC3, TNIP2, NKIRAS2 |
|  | GO:0043488~regulation of mRNA stability | 8 | 0.015 | EIF4G1, PSMB4, CARHSP1, UBC, HSPB1, SCGB1A1, HSPA8, SAMD4A |
|  | GO:0000187~activation of MAPK activity | 8 | 0.018 | IRAK2, CXCL17, TNF, C5AR1, UBC, CHRNA7, TLR4, INSR |
|  | GO:0098656~anion transmembrane transport | 7 | 9E-05 | LRRC8A, LRRC8D, ABCC10, SLC4A4, ABCC8, ABCC5, VDAC1 |
|  | GO:0031663~lipopolysaccharide-mediated signaling pathway | 6 | 0.001 | IRAK2, TNF, HCK, NFKBIA, TLR4, NOS3 |
|  | GO:0045429~positive regulation of nitric oxide biosynthetic process | 6 | 0.004 | OPRM1, TNF, INS, PKD2, TLR4, INSR |
|  | GO:0071363~cellular response to growth factor stimulus | 6 | 0.005 | PDGFB, RIPK1, TH, MAPK7, INSR, CTNNB1 |
|  | GO:0046330~positive regulation of JNK cascade | 6 | 0.023 | DUSP19, TNF, TAOK2, RIPK1, TLR4, TPD52L1 |
|  | GO:0006006~glucose metabolic process | 6 | 0.026 | TNF, INS, FBN1, GAA, FABP5, CACNA1A |
|  | GO:0006816~calcium ion transport | 6 | 0.042 | PKD2, CCL8, CHRNA7, TRPV6, CALCRL, CACNA1A |
|  | GO:0042147~retrograde transport, endosome to Golgi | 6 | 0.029 | UBE2O, GBF1, DENND2A, CLTC, VPS26B, DCTN1 |
|  | GO:0035666~TRIF-dependent toll-like receptor signaling pathway | 5 | 0.005 | RIPK1, TICAM2, UBC, TLR4, BIRC3 |
|  | GO:1902176~negative regulation of oxidative stress-induced intrinsic apoptotic signaling pathway | 4 | 0.005 | BAG5, INS, HSPB1, MAPK7 |
|  | GO:0032270~positive regulation of cellular protein metabolic process | 4 | 0.006 | EIF4G1, AGTR1, INS, NFKBIA |
|  | GO:0050995~negative regulation of lipid catabolic process | 4 | 0.007 | CRTC3, TNF, INS, APOC3 |
|  | GO:0032715~negative regulation of interleukin-6 production | 4 | 0.032 | TNF, CHRNA7, TLR4, FOXP3 |
|  | GO:0030449~regulation of complement activation | 4 | 0.039 | C7, C5AR1, C3, CD46 |
|  | GO:0010803~regulation of tumor necrosis factor-mediated signaling pathway | 4 | 0.039 | TNF, RIPK1, UBC, BIRC3 |
|  | GO:0000722~telomere maintenance via recombination | 4 | 0.045 | PRIM1, POLD1, POLE, SMC6 |
|  | GO:0042116~macrophage activation | 3 | 0.025 | CRTC3, TLR4, FOXP1 |
|  | GO:0010939~regulation of necrotic cell death | 3 | 0.035 | RIPK1, UBC, BIRC3 |
|  | GO:0007009~plasma membrane organization | 3 | 0.041 | MTSS1, FAT4, BAIAP2 |
|  | GO:0002756~MyD88-independent toll-like receptor signaling pathway | 3 | 0.041 | TICAM2, UBC, TLR4 |
|  | GO:0007398~ectoderm development | 3 | 0.041 | KRT6B, EPB41L5, CTNNB1 |
|  | GO:0006820~anion transport | 3 | 0.047 | LRRC8A, SLC37A3, VDAC1 |
| **P150 up-regulated** | GO:0007165~signal transduction | 44 | 0.008 | FGF18, CCL2, GABRB3, HINT1, NR3C2, KLRK1, PDE3B, DEK, RANGAP1, CXCL11, CNOT7, CD2AP, CCL26, PGR, TNFRSF1A, FGA, UNC5B, ANK3, ZYX, INPP5D, MX1, AGAP3, PIK3R2, OR5I1, TNFSF4, MAP2K1, RAN, MAP2K3, STK4, RALGDS, RPS6KL1, TNFRSF10A, RASSF6, TNFRSF10D, ROR2, JAK2, WIF1, STMN1, SYNGAP1, ARAP1, SRGAP1, IGFBP4, BCAR3, DNM2 |
|  | GO:0043547~positive regulation of GTPase activity | 23 | 0.031 | FGF18, CCL2, ARHGEF1, GNAO1, HPS4, GRHL3, RANGAP1, RGS16, ITSN1, RALGDS, CCL26, SH2D3C, NDEL1, GRIN2B, GIT2, JAK2, SYNGAP1, AGAP3, ARAP1, SRGAP1, ELMOD1, BCAR3, PIK3R2 |
|  | GO:0008283~cell proliferation | 18 | 0.012 | CDV3, ARHGEF1, CDK9, RPL23A, CDK7, LIG4, DAZAP1, ZFP36L1, PROK2, IFNAR2, GFI1B, SETMAR, H3F3B, RAP1B, BHLHE41, DDX41, IGFBP4, THPO |
|  | GO:0006413~translational initiation | 16 | 2E-06 | RPL17, RPL27A, RPL23A, RPS4X, RPS2, RPL39, RPS26, EIF4G2, RPL30, RPS29, RPL9, EIF4H, RPL10, RPL12, RPS21, RPS27A |
|  | GO:0051301~cell division | 16 | 0.034 | CCNT2, ANAPC5, TSG101, RAN, LIG1, CETN2, UBE2I, CDK7, LIG4, LATS1, CD2AP, MAD2L1, SEH1L, NCAPG2, MCMBP, CDK20 |
|  | GO:0000184~nuclear-transcribed mRNA catabolic process, nonsense-mediated decay | 15 | 2E-06 | RPL17, RPL27A, RPL23A, RPS4X, RPL39, RPS2, RPS26, RPL30, RPS29, DCP1B, RPL9, RPL10, RPL12, RPS21, RPS27A |
|  | GO:0016032~viral process | 15 | 0.021 | NUP133, CRTC2, RAN, UBE2I, KAT5, CBX5, WDR48, TNFRSF1A, ULBP3, SEH1L, EIF4H, MDM2, HSPD1, TCEB1, ZYX |
|  | GO:0006614~SRP-dependent cotranslational protein targeting to membrane | 14 | 7E-07 | RPL17, RPS26, RPL30, RPS29, RPL9, RPL27A, RPL10, RPL23A, RPL12, RPS4X, RPS2, RPL39, RPS21, RPS27A |
|  | GO:0001525~angiogenesis | 14 | 0.005 | SAT1, FGF18, EMCN, CCL2, PDE3B, ESM1, MCAM, PROK2, WARS, UNC5B, FAP, HOXA7, SETD2, JAM3 |
|  | GO:0000398~mRNA splicing, via spliceosome | 13 | 0.011 | SNRPA1, PRPF31, RBM41, ZMAT5, SRSF11, TRA2A, BUD31, SF3B1, PRPF8, RSRC1, DDX41, CSTF1, SNRPE |
|  | GO:0016925~protein sumoylation | 11 | 8E-04 | SUMO2, NUP133, EID3, SEH1L, MDC1, PIAS3, CETN2, MDM2, RANGAP1, UBE2I, RAD52 |
|  | GO:0042127~regulation of cell proliferation | 11 | 0.02 | TNFRSF10A, SAT1, TYK2, MAFG, TNFRSF1A, TNFRSF10D, JTB, CFDP1, JAK2, CXCL11, NKX2-3 |
|  | GO:0007050~cell cycle arrest | 10 | 0.01 | EIF4G2, CDKN1A, MAP2K1, TSG101, IRF6, RRAGA, CDK7, MLF1, TP53INP1, BARD1 |
|  | GO:0042787~protein ubiquitination involved in ubiquitin-dependent protein catabolic process | 10 | 0.016 | MAD2L1, ANAPC5, KLHL25, MDM2, TCEB1, RCHY1, ITCH, RPS27A, HECTD1, RNF111 |
|  | GO:0070911~global genome nucleotide-excision repair | 7 | 1E-04 | RAD23B, SUMO2, GTF2H4, CETN2, UBE2I, RPS27A, RNF111 |
|  | GO:0006283~transcription-coupled nucleotide-excision repair | 7 | 0.011 | HMGN1, RPA2, LIG1, GTF2H4, CDK7, XRCC1, RPS27A |
|  | GO:0050729~positive regulation of inflammatory response | 6 | 0.037 | TNFRSF1A, CCL2, TNFSF4, JAK2, HSPD1, CCL26 |
|  | GO:0006294~nucleotide-excision repair, preincision complex assembly | 6 | 7E-04 | RAD23B, RPA2, GTF2H4, CETN2, CDK7, RPS27A |
|  | GO:0075733~intracellular transport of virus | 6 | 0.009 | NMT2, NUP133, TSG101, SEH1L, RAN, RPS27A |
|  | GO:0006303~double-strand break repair via nonhomologous end joining | 6 | 0.021 | MDC1, SETMAR, LIG4, KAT5, UIMC1, BARD1 |
|  | GO:0006302~double-strand break repair | 6 | 0.026 | APLF, LIG1, LIG4, KAT5, RAD52, UIMC1 |
|  | GO:0034976~response to endoplasmic reticulum stress | 6 | 0.041 | UFM1, TMX3, PDIA6, ANKS4B, PDIA4, PIK3R2 |
|  | GO:0006297~nucleotide-excision repair, DNA gap filling | 5 | 0.003 | RPA2, LIG1, LIG4, XRCC1, RPS27A |
|  | GO:0006259~DNA metabolic process | 5 | 0.004 | NME1, RAN, LIG1, IGFBP4, TK1 |
|  | GO:0007259~JAK-STAT cascade | 5 | 0.008 | IFNAR2, CCL2, STAT5A, SOCS1, JAK2 |
|  | GO:0006284~base-excision repair | 5 | 0.011 | HMGB1, RPA2, LIG1, SIRT6, XRCC1 |
|  | GO:0000717~nucleotide-excision repair, DNA duplex unwinding | 4 | 0.017 | RAD23B, GTF2H4, CETN2, RPS27A |
|  | GO:0046677~response to antibiotic | 4 | 0.046 | CCL2, RSRC1, MDM2, JAK2 |
|  | GO:0033629~negative regulation of cell adhesion mediated by integrin | 3 | 0.016 | MUC1, ACER2, PDE3B |
|  | GO:0000012~single strand break repair | 3 | 0.016 | APLF, LIG4, XRCC1 |
|  | GO:0051103~DNA ligation involved in DNA repair | 3 | 0.025 | HMGB1, LIG1, LIG4 |
|  | GO:0006098~pentose-phosphate shunt | 3 | 0.03 | PGM2, TPI1, PGD |
|  | GO:0006107~oxaloacetate metabolic process | 3 | 0.036 | STAT5A, MDH2, PCK1 |
| **P150 down-regulated** | GO:0007155~cell adhesion | 13 | 0.02 | PLXNC1, EGFL7, CCR1, PCDH10, CLSTN1, CX3CR1, ICAM3, ITGAD, CD151, CXCL12, CDH5, APLP1, AOC3 |
|  | GO:0010629~negative regulation of gene expression | 6 | 0.036 | WWP2, CRYAB, CCR1, YY1, ACACB, CD28 |
|  | GO:0042776~mitochondrial ATP synthesis coupled proton transport | 4 | 0.003 | ATP5D, ATP5E, ATP5B, ATP5G1 |
|  | GO:0006754~ATP biosynthetic process | 4 | 0.007 | ATP5D, ATP5E, ATP5B, ATP5G1 |
|  | GO:0015986~ATP synthesis coupled proton transport | 3 | 0.034 | ATP5D, ATP5B, ATP5G1 |
|  | GO:0071108~protein K48-linked deubiquitination | 3 | 0.04 | USP8, USP34, OTUB2 |
|  | GO:0031297~replication fork processing | 3 | 0.05 | EME1, RTEL1, RAD51 |
