# Supplementary material for: Calves Infected with Virulent and Attenuated Mycoplasma bovis Strains Have Upregulated Th17 Inflammatory and Th1 Protective Responses, Respectively
Source: Genes (Basel). 2019 Aug 28;10(9):656. doi: 10.3390/genes10090656 (PMC6770603; doi:10.3390/genes10090656)
Supplement: Supplementary file 1 [file genes-10-00656-s001.zip › supplement/Table S5.docx]

| **Gene**  **Symbol** | **Genbank**  **Accession** | **Probe**  **Name** | **Microarray** | | | |  | **q-PCR** | | | |
| --- | --- | --- | --- | --- | --- | --- | --- | --- | --- | --- | --- |
|  |  |  | **P1** | | **P150** | |  | **P1** | | **P150** | |
|  |  |  | **Fold Chang** | **SD** | **Fold Chang** | **SD** |  | **Fold Chang** | **SD** | **Fold Chang** | **SD** |
| TRPV4 | NM_001192385 | A_73_108024 | 9.75 | 0.20 | 3.46 | 0.06 |  | 4.88 | 1.10 | 2.30 | 0.54 |
| NOD1 | NM_001256563 | A_73_103196 | 11.36 | 0.63 | 3.47 | 0.32 |  | 5.60 | 0.81 | 3.17 | 0.73 |
| PIK3CB | NM_001206047 | A_73_P316391 | 3.88 | 0.06 | 1.95 | 0.04 |  | 2.73 | 0.62 | 2.31 | 0.25 |
| SYK | NM_001037465 | A_73_P366781 | 4.85 | 0.20 | 1.17 | 0.05 |  | 10.95 | 3.50 | 1.50 | 0.37 |
| IL17D | XM_002691866 | A_73_P077271 | 3.62 | 0.07 | 1.66 | 0.18 |  | 6.29 | 1.84 | 1.85 | 0.41 |
| IL21R | NM_001193179 | A_73_109180 | 2.04 | 0.02 | 1.25 | 0.10 |  | 2.38 | 0.72 | 1.12 | 0.12 |
| IL23R | NM_001127172 | A_73_117664 | 3.61 | 0.42 | 1.02 | 0.05 |  | 3.56 | 0.87 | 1.56 | 0.19 |
| MDM2 | NM_001099107 | A_73_116639 | 1.69 | 0.08 | 2.98 | 0.12 |  | 0.81 | 0.24 | 4.05 | 1.28 |
| TLR4 | NM_174198 | A_73_105309 | 0.46 | 0.02 | 0.66 | 0.03 |  | 0.84 | 0.23 | 1.35 | 0.33 |
