# Supplementary material for: Calves Infected with Virulent and Attenuated Mycoplasma bovis Strains Have Upregulated Th17 Inflammatory and Th1 Protective Responses, Respectively
Source: Genes (Basel). 2019 Aug 28;10(9):656. doi: 10.3390/genes10090656 (PMC6770603; doi:10.3390/genes10090656)
Supplement: Supplementary file 1 [file genes-10-00656-s001.zip › supplement/Table S6.docx]

| **Genes of P1 vs NC group** | **FC (P1/NC)** | **FC (P150/NC)** | **FC (P1/P150)** | **Genbank**  **Accession** | **Gene Name** | **Pathways** | **Immune pathways** |
| --- | --- | --- | --- | --- | --- | --- | --- |
| SYK | 4.84 | 1.17 | 4.13 | NM_001037465 | spleen tyrosine kinase | 04064: NF-kappa B signaling pathway  04072: Phospholipase D signaling pathway  04151: PI3K-Akt signaling pathway  04611: Platelet activation  04650: Natural killer cell mediated cytotoxicity  04662: B cell receptor signaling pathway  04666: Fc gamma R-mediated phagocytosis  04659: Th17 cell differentiation | 04611: Platelet activation  04625: C-type lectin receptor signaling pathway  04650: Natural killer cell mediated cytotoxicity  04662: B cell receptor signaling pathway  04664: Fc epsilon RI signaling pathway  04666: Fc gamma R-mediated phagocytosis |
| TP53 | 2.54 | 0.78 | 3.25 | NM_174201 | tumor protein p53 | 04010: MAPK signaling pathway  04110: Cell cycle  04115: p53 signaling pathway  04137: Mitophagy - animal  04151: PI3K-Akt signaling pathway  04210: Apoptosis  04218: Cellular senescence |  |
| IL6 | 2.39 | 0.73 | 3.27 | NM_173923 | interleukin 6 (interferon, beta 2) | 04060: Cytokine-cytokine receptor interaction  04066: HIF-1 signaling pathway  04068: FoxO signaling pathway  04151: PI3K-Akt signaling pathway  04218: Cellular senescence  04620: Toll-like receptor signaling pathway  04621: NOD-like receptor signaling pathway  04623: Cytosolic DNA-sensing pathway  04630: Jak-STAT signaling pathway  04640: Hematopoietic cell lineage  04657: IL-17 signaling pathway  04659: Th17 cell differentiation  04668: TNF signaling pathway  04672: Intestinal immune network for IgA production | 04640: Hematopoietic cell lineage  04620 :Toll-like receptor signaling pathway  04621: NOD-like receptor signaling pathway  04623: Cytosolic DNA-sensing pathway  04625: C-type lectin receptor signaling pathway  04659: Th17 cell differentiation  04657: IL-17 signaling pathway  04672: Intestinal immune network for IgA production |
| STAT3 | 3.42 | 1.22 | 2.79 | NM_001012671 | signal transducer and activator of transcription 3 (acute-phase response factor) | 01521: EGFR tyrosine kinase inhibitor resistance  04062: Chemokine signaling pathway  04066: HIF-1 signaling pathway  04068: FoxO signaling pathway  04217 : Necroptosis  04550: Signaling pathways regulating pluripotency of stem cells  04630: Jak-STAT signaling pathway  04659: Th17 cell differentiation | 04659: Th17 cell differentiation 04062: Chemokine signaling pathway |
| ATP5B | 11.09 | 0.98 | 11.37 | NM_175796 | ATP synthase, H+ transporting, mitochondrial F1 complex, beta polypeptide | 00190: Oxidative phosphorylation/01100: Metabolic pathways |  |
| GSK3B | 3.31 | 0.64 | 5.13 | NM_001101310 | glycogen synthase kinase 3 beta | 01521: EGFR tyrosine kinase inhibitor resistance  04062: Chemokine signaling pathway  04110: Cell cycle  04150: mTOR signaling pathway  04151: PI3K-Akt signaling pathway  04510: Focal adhesion  04550: Signaling pathways regulating pluripotency of stem cells  04657: IL-17 signaling pathway  04660: T cell receptor signaling pathway  04662: B cell receptor signaling pathway | 04660: T cell receptor signaling pathway  04657: IL-17 signaling pathway  04662: B cell receptor signaling pathway  04062: Chemokine signaling pathway |
| ACTG1 | 2.64 | 1.21 | 2.18 | NM_001033618 | actin, gamma 1 | 04015: Rap1 signaling pathway  04145: Phagosome  04210: Apoptosis  04510: Focal adhesion  04520: Adherens junction  04530: Tight junction  04611: Platelet activation  04670: Leukocyte transendothelial migration  04810: Regulation of actin cytoskeleton  05100: Bacterial invasion of epithelial cells | 04611: Platelet activation  04670: Leukocyte transendothelial migration |
| IL17D | 3.62 | 1.65 | 2.20 | NM_001193179 | interleukin 17D | 04060: Cytokine-cytokine receptor interaction  04625: C-type lectin receptor signaling pathway  04630: Jak-STAT signaling pathway  04657: IL-17 signaling pathway  04659: Th17 cell differentiation | 04625: C-type lectin receptor signaling pathway  04657: IL-17 signaling pathway  04659: Th17 cell differentiation |
| IL21R | 2.04 | 1.25 | 1.63 | NM_001193179 | interleukin 21 receptor | 04060: Cytokine-cytokine receptor interaction  04630: Jak-STAT signaling pathway  04659: Th17 cell differentiation  05321: Inflammatory bowel disease (IBD) | 04659: Th17 cell differentiation |
| IL23R | 3.59 | 1.02 | 3.53 | NM_001127172 | interleukin 23 receptor | 04060: Cytokine-cytokine receptor interaction  04630: Jak-STAT signaling pathway  04659: Th17 cell differentiation  05200: Pathways in cancer  05321: Inflammatory bowel disease (IBD) | 04659: Th17 cell differentiation |
| JAK1 | 10.65 | 0.81 | 13.12 | NM_001206534 | Janus kinase 1 | 01521: EGFR tyrosine kinase inhibitor resistance  04151: PI3K-Akt signaling pathway  04217: Necroptosis  04380: Osteoclast differentiation  04550: Signaling pathways regulating pluripotency of stem cells  04621: NOD-like receptor signaling pathway  04630: Jak-STAT signaling pathway  04658: Th1 and Th2 cell differentiation  04659: Th17 cell differentiation  05140: Leishmaniasis  05145: Toxoplasmosis  05152: Tuberculosis  05160: Hepatitis C  05161: Hepatitis B  05162: Measles  05163: Human cytomegalovirus infection  05164: Influenza A  05165: Human papillomavirus infection  05166: HTLV-I infection  05167: Kaposi's sarcoma-associated herpesvirus infection  05168: Herpes simplex infection  05169: Epstein-Barr virus infection  05200: Pathways in cancer  05203: Viral carcinogenesis  05212: Pancreatic cancer | 04621: NOD-like receptor signaling pathway  04658: Th1 and Th2 cell differentiation  04659: Th17 cell differentiation |
| RORB | 8.00 | 0.91 | 8.81 | NM_001192658 | RAR-related orphan receptor B | 04710: Circadian rhythm |  |
| GATA3 | 1.62 | 0.95 | 1.72 | NM_001076804 | GATA binding protein 3 | 04658: Th1 and Th2 cell differentiation  04659: Th17 cell differentiation  04928: Parathyroid hormone synthesis, secretion and action | 04658: Th1 and Th2 cell differentiation  04659: Th17 cell differentiation |
| IL1RL1 | 2.95 | 1.47 | 2.01 | NM_001206302 | interleukin 1 receptor-like 1 | [04060](https://www.kegg.jp/kegg-bin/show_pathway?ko04060+K05171): Cytokine-cytokine receptor interaction |  |
| UBC | 0.29 | 0.61 | 0.47 | NM_001206307 | ubiquitin C | [03320](http://www.kegg.jp/kegg-bin/show_pathway?ko03320+K08770):PPAR signaling pathway |  |
| TNF | 0.35 | 0.56 | 0.62 | NM_173966 | tumor necrosis factor | 01523: Antifolate resistance  04010: MAPK signaling pathway  04060: Cytokine-cytokine receptor interaction  04064: NF-kappa B signaling pathway  04071: Sphingolipid signaling pathway  04150: mTOR signaling pathway  04210: Apoptosis  04217: Necroptosis  04350: TGF-beta signaling pathway  04612: Antigen processing and presentation  04620: Toll-like receptor signaling pathway  04621: NOD-like receptor signaling pathway  04622: RIG-I-like receptor signaling pathway  04650: Natural killer cell mediated cytotoxicity  04657: IL-17 signaling pathway  04660: T cell receptor signaling pathway  04664: Fc epsilon RI signaling pathway  04668: TNF signaling pathway | 04640: Hematopoietic cell lineage  04620: Toll-like receptor signaling pathway  04621: NOD-like receptor signaling pathway  04622: RIG-I-like receptor signaling pathway  04625: C-type lectin receptor signaling pathway  04650: Natural killer cell mediated cytotoxicity  04612: Antigen processing and presentation  04660: T cell receptor signaling pathway  04657: IL-17 signaling pathway  04664: Fc epsilon RI signaling pathway |
| TLR4 | 0.46 | 0.66 | 0.70 | NM_174198 | toll-like receptor 4 | 04064: NF-kappa B signaling pathway  04066: HIF-1 signaling pathway  04151: PI3K-Akt signaling pathway  04145: Phagosome  04217: Necroptosis  04620: Toll-like receptor signaling pathway  04621: NOD-like receptor signaling pathway | 04620: Toll-like receptor signaling pathway  04621: NOD-like receptor signaling pathway |
| INS | 0.19 | 0.54 | 0.35 | NM_001185126 | insulin | 04010: MAPK signaling pathway  04014: Ras signaling pathway  04015: Rap1 signaling pathway  04022: cGMP-PKG signaling pathway  04066: HIF-1 signaling pathway  04068: FoxO signaling pathway  04072: Phospholipase D signaling pathway  04140: Autophagy - animal  04150: mTOR signaling pathway  04151: PI3K-Akt signaling pathway  04152: AMPK signaling pathway |  |
| HSPA8 | 0.44 | 0.70 | 0.63 | NM_174345 | heat shock 70kDa protein 8 | 03040: Spliceosome  04010: MAPK signaling pathway  04141: Protein processing in endoplasmic reticulum  04144: Endocytosis  04612: Antigen processing and presentation | 04612: Antigen processing and presentation |
| NFKBIA | 0.37 | 0.59 | 0.62 | NM_001045868 | nuclear factor of kappa light polypeptide gene enhancer in B-cells inhibitor, alpha | 04024: cAMP signaling pathway  04062: Chemokine signaling pathway  04064: NF-kappa B signaling pathway  04210: Apoptosis  04620: Toll-like receptor signaling pathway  04621: NOD-like receptor signaling pathway  04622: RIG-I-like receptor signaling pathway  04657: IL-17 signaling pathway  04658: Th1 and Th2 cell differentiation  04659: Th17 cell differentiation  04660: T cell receptor signaling pathway  04662: B cell receptor signaling pathway  04668: TNF signaling pathway | 04620: Toll-like receptor signaling pathway  04621: NOD-like receptor signaling pathway  04622: RIG-I-like receptor signaling pathway  04623: Cytosolic DNA-sensing pathway  04625: C-type lectin receptor signaling pathway  04660: T cell receptor signaling pathway  04658: Th1 and Th2 cell differentiation  04659: Th17 cell differentiation  04657: IL-17 signaling pathway  04662: B cell receptor signaling pathway  04062: Chemokine signaling pathway |
| INSR | 0.21 | 0.70 | 0.30 | XM_002688832 | insulin receptor | 04010: MAPK signaling pathway  04014: Ras signaling pathway  04015: Rap1 signaling pathway  04022: cGMP-PKG signaling pathway  04066: HIF-1 signaling pathway  04068: FoxO signaling pathway  04072: Phospholipase D signaling pathway  04150: mTOR signaling pathway  04151: PI3K-Akt signaling pathway  04152: AMPK signaling pathway |  |
| BIRC3 | 0.49 | 0.90 | 0.54 | NM_001035293 | baculoviral IAP repeat containing 3 | 04064: NF-kappa B signaling pathway  04120: Ubiquitin mediated proteolysis  04210: Apoptosis  04215: Apoptosis - multiple species  04217: Necroptosis  04510: Focal adhesion  04621: NOD-like receptor signaling pathway  04668: TNF signaling pathway | 04621: NOD-like receptor signaling pathway |
| NOS3 | 0.43 | 1.18 | 0.36 | NM_181037 | nitric oxide synthase 3 | 00220: Arginine biosynthesis  00330: Arginine and proline metabolism  01100: Metabolic pathways  04020: Calcium signaling pathway  04022: cGMP-PKG signaling pathway  04066: HIF-1 signaling pathway  04071: Sphingolipid signaling pathway  04151: PI3K-Akt signaling pathway  04370: VEGF signaling pathway  04611: Platelet activation  04915: Estrogen signaling pathway  04921: Oxytocin signaling pathway  04926: Relaxin signaling pathway  04931: Insulin resistance  04933: AGE-RAGE signaling pathway in diabetic complications  05418: Fluid shear stress and atherosclerosis | 04611: Platelet activation |
| FOXP3 | 0.40 | 0.70 | 0.51 | BC149282 | forkhead box P3 | [04659](https://www.kegg.jp/kegg-bin/show_pathway?ko04659+K10163): Th17 cell differentiation  05321: Inflammatory bowel disease (IBD) | [04659](https://www.kegg.jp/kegg-bin/show_pathway?ko04659+K10163): Th17 cell differentiation |

| **Genes of P150 vs NC group** | **FC (P1/NC)** | **FC (P150/NC)** | **FC (P150/P1)** | **Genbank**  **Accession** | **Gene**  **Name** | **Pathways** | **Immune pathways** |
| --- | --- | --- | --- | --- | --- | --- | --- |
| UBE2I | 1.30 | 2.18 | 1.68 | NM_001099372 | ubiquitin-conjugating enzyme E2I | 03013: RNA transport  04064: NF-kappa B signaling pathway  04120: Ubiquitin mediated proteolysis |  |
| RAD23B | 1.05 | 4.03 | 3.84 | NM_001046310 | RAD23 homolog B | 03420: Nucleotide excision repair  04141: Protein processing in endoplasmic reticulum |  |
| GTF2H4 | 1.03 | 5.00 | 4.86 | NM_001101057 | general transcription factor IIH, polypeptide 4 | 03022: Basal transcription factors  03420: Nucleotide excision repair  05203: Viral carcinogenesis |  |
| SOCS1 | 2.00 | 2.61 | 1.31 | XM_864316 | suppressor of cytokine signaling 1 | 04120: Ubiquitin mediated proteolysis  04630: Jak-STAT signaling pathway  04910: Insulin signaling pathway |  |
| MDM2 | 1.69 | 2.97 | 1.76 | NM_001099107 | Mdm2, p53 E3 ubiquitin protein ligase homolog | 04068: FoxO signaling pathway  04110: Cell cycle  04115: p53 signaling pathway  04120: Ubiquitin mediated proteolysis  04144: Endocytosis  04151: PI3K-Akt signaling pathway |  |
| TCEB1 | 1.06 | 2.24 | 2.12 | NM_001046493 | transcription elongation factor B (SIII), polypeptide 1 | 04066: HIF-1 signaling pathway  04120: Ubiquitin mediated proteolysis |  |
| CDK7 | 0.98 | 3.53 | 3.61 | NM_001075715 | cyclin-dependent kinase 7 | 03022: Basal transcription factors  03420: Nucleotide excision repair  04110: Cell cycle |  |
| ITCH | 1.81 | 4.49 | 2.48 | NM_001082428 | itchy E3 ubiquitin protein ligase homolog | 04120: Ubiquitin mediated proteolysis  04144: Endocytosis  04668: TNF signaling pathway |  |
| UBE2D3 | 1.93 | 2.34 | 1.22 | NM_001075135 | ubiquitin-conjugating enzyme E2D 3 | 04120: Ubiquitin mediated proteolysis  04141: Protein processing in endoplasmic reticulum |  |
| UBE2E3 | 1.71 | 2.88 | 1.68 | NM_001079783 | ubiquitin-conjugating enzyme E2E 3 | 04120: Ubiquitin mediated proteolysis |  |
| RCHY1 | 0.96 | 3.20 | 3.32 | NM_001083754 | ring finger and CHY zinc finger domain containing 1 | 04115: p53 signaling pathway  04120: Ubiquitin mediated proteolysis |  |
| RORC | 0.66 | 0.43 | 1.54 | NM_001083451 | RAR-related orphan receptor C | 04659: Th17 cell differentiation  04710: Circadian rhythm  05321: Inflammatory bowel disease (IBD) | 04659: Th17 cell differentiation  05321: Inflammatiory bowel disease (IBD) |
