# Supplementary material for: Calves Infected with Virulent and Attenuated Mycoplasma bovis Strains Have Upregulated Th17 Inflammatory and Th1 Protective Responses, Respectively
Source: Genes (Basel). 2019 Aug 28;10(9):656. doi: 10.3390/genes10090656 (PMC6770603; doi:10.3390/genes10090656)
Supplement: Supplementary file 1 [file genes-10-00656-s001.zip › supplement/Table S7.docx]

| **Abbreviation** | **Full name** |
| --- | --- |
| ACTG1 | actin gamma 1 |
| AMOT | angiomotin |
| ANOVA | analysis of variance |
| ARRB2 | arrestin, beta 2 |
| ATP5B | ATP synthase, H+ transporting, mitochondrial F1 complex, beta polypeptide |
| BALF | bronchoalveolar lavage fluid |
| BIRC3 | baculoviral IAP repeat containing 3 |
| BMP4 | bone morphogenetic protein 4 |
| BVDV | bovine viral diarrhea virus |
| CCL2 | chemokine (C-C motif) ligand 2 |
| CDK7 | cyclin-dependent kinase 7 |
| CETN2 | centrin, EF-hand protein, 2 |
| CFU | colony forming units |
| CHRNA7 | cholinergic receptor, nicotinic, alpha 7 |
| CRTC3 | CREB regulated transcription coactivator 3 |
| DEGs | differentially expressed genes |
| dpi | days post inoculation |
| EIF4G1 | eukaryotic translation initiation factor 4 gamma, 1 |
| FOXP3 | forkhead box P3 |
| GATA3 | GATA binding protein 3 box P3 |
| GO | Gene Ontology |
| GSK3B | glycogen synthase kinase 3 beta |
| GTF2H4 | general transcription factor IIH, polypeptide 4 |
| H&E | ematoxylin and eosin |
| HAX1 | HCLS1 associated protein X-1 |
| HCLS1 | hematopoietic cell-specific Lyn substrate 1 |
| HSPA8 | heat shock 70kDa protein 8 |
| IBRV | infectious bovine rhinotracheitis virus |
| IFNG | interferon, gamma |
| IL | interleukin |
| IL-1RL1 | interleukin 1 receptor-like 1 |
| INS | insulin |
| INSR | insulin receptor |
| IRAK2 | interleukin-1 receptor-associated kinase 2 |
| ITCH | itchy E3 ubiquitin protein ligase |
| JAK | Janus kinase |
| KEGG | Kyoto Encyclopedia of Genes and Genomes |
| LIG1 | ligase I, DNA, ATP-dependent |
| LIG4 | ligase IV, DNA, ATP-dependent |
| *M. bovis* | *Mycoplasma bovis* |
| MDM2 | P53 E3 ubiquitin protein ligase homolog |
| NFKBIA | nuclear factor of kappa light polypeptide gene enhancer in B-cells inhibitor, alpha |
| NOD1 | nucleotide-binding oligomerization domain containing 1 |
| NOS3 | nitric oxide synthase 3 (endothelial cell) |
| PBMCs | peripheral blood mononuclear cells |
| PCR | polymerase chain reaction |
| PIK3CB | phosphoinositide-3-kinase, catalytic, beta polypeptide |
| PPI | protein-protein interaction |
| PPLO | pleuropneumonia-like organism |
| qPCR | quantitative real-time polymerase chain reaction |
| RAD23B | RAD23 homolog B (S. cerevisiae) |
| RAF1 | v-raf-1 murine leukemia viral oncogene homolog 1 |
| RAMP2 | receptor (G protein-coupled) activity modifying protein 2 |
| RAN | RAN, member RAS oncogene family |
| RBM5 | RNA binding motif protein 5 |
| RCHY1 | ring finger and CHY zinc finger domain containing 1 |
| RIPK | receptor-interacting serine-threonine kinase 2 |
| ROR | retinoid-related orphan receptors |
| RPA2 | replication protein A2, 32kDa |
| RPL23A | ribosomal protein L23a |
| RPS27A | ribosomal protein S27a |
| SEH1L | SEH1-like |
| SGK1 | serum/glucocorticoid regulated kinase 1 |
| SOCS1 | suppressor of cytokine signaling 1 |
| STAT3 | signal transducer and activator of transcription 3 |
| STRING | Search Tool for the Retrieval of Interacting Genes |
| SYK | spleen tyrosine kinase |
| TCEB1 | transcription elongation factor B (SIII), polypeptide 1 |
| Teff17 | effector Th17 |
| TICAM2 | toll-like receptor adaptor molecule 2 |
| TLR | toll-like receptor |
| TNF | tumor necrosis factor |
| TNFRSF1A | tumor necrosis factor receptor superfamily, member 1A |
| TP53 | tumor protein p53 |
| Treg17 | regulatory Th17 |
| TRPV4 | transient receptor potential cation channel, subfamily V, member 4 |
| UBC | ubiquitin C |
| UBE | ubiquitin-conjugating enzyme E |
| VDAC1 | voltage-dependent anion channel 1 |
