# Supplementary figures and images for: Calves Infected with Virulent and Attenuated Mycoplasma bovis Strains Have Upregulated Th17 Inflammatory and Th1 Protective Responses, Respectively
Source: Genes (Basel). 2019 Aug 28;10(9):656. doi: 10.3390/genes10090656 (PMC6770603; doi:10.3390/genes10090656)

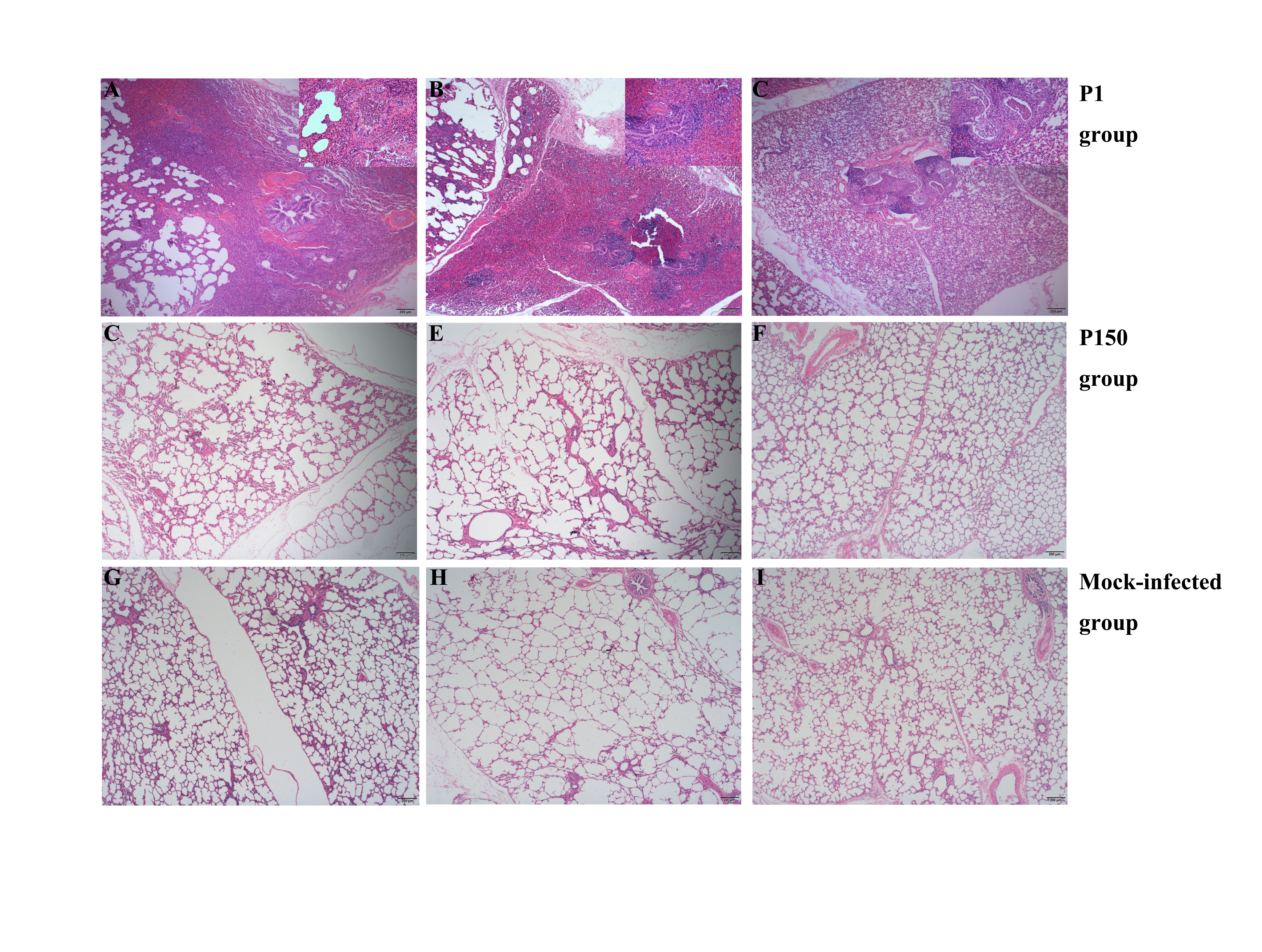

Supplement: Supplementary file 1 [file genes-10-00656-s001.zip › supplement/Figure S2.tif]

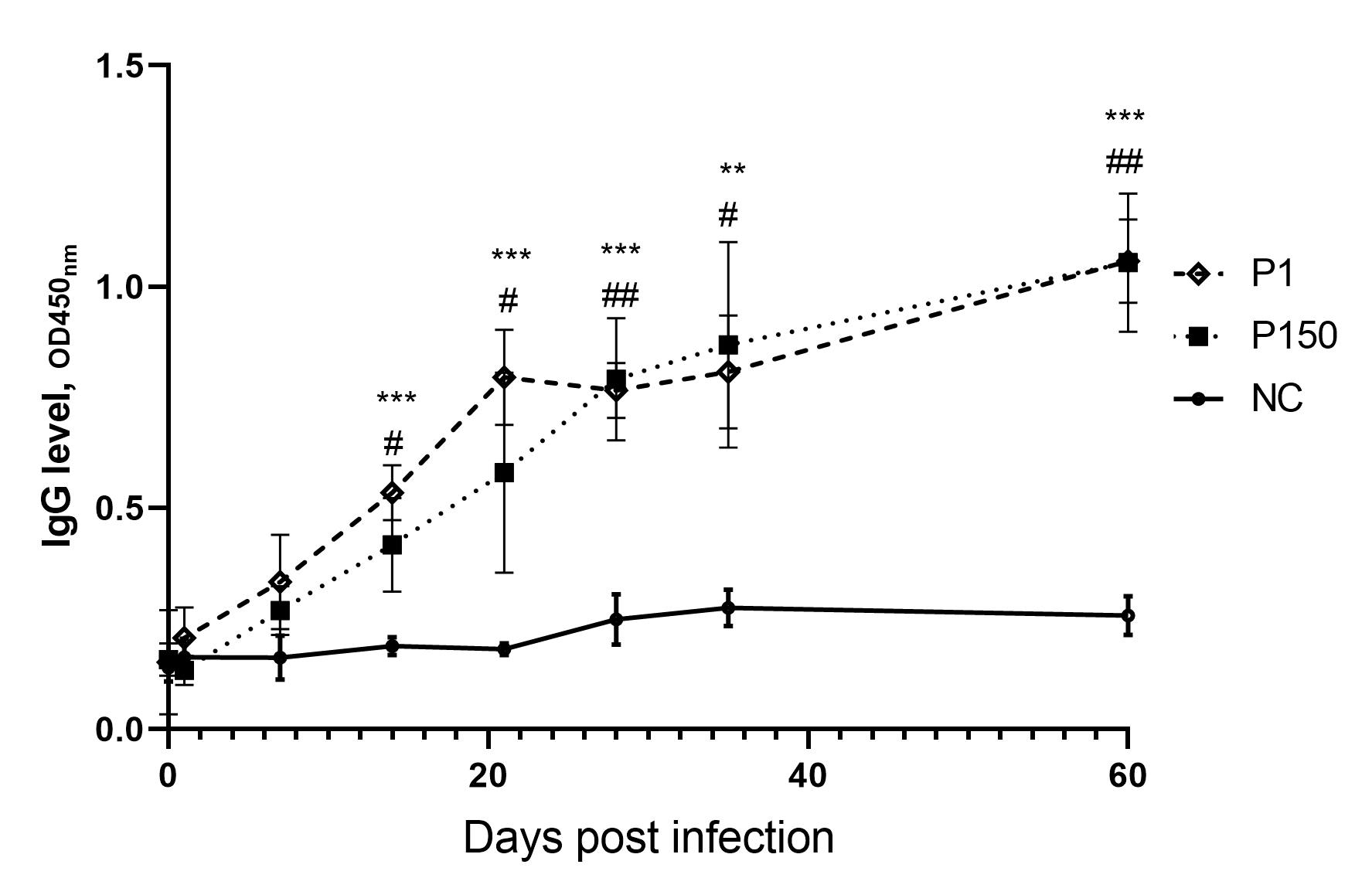

Supplement: Supplementary file 1 [file genes-10-00656-s001.zip › supplement/Figure S3.tif]

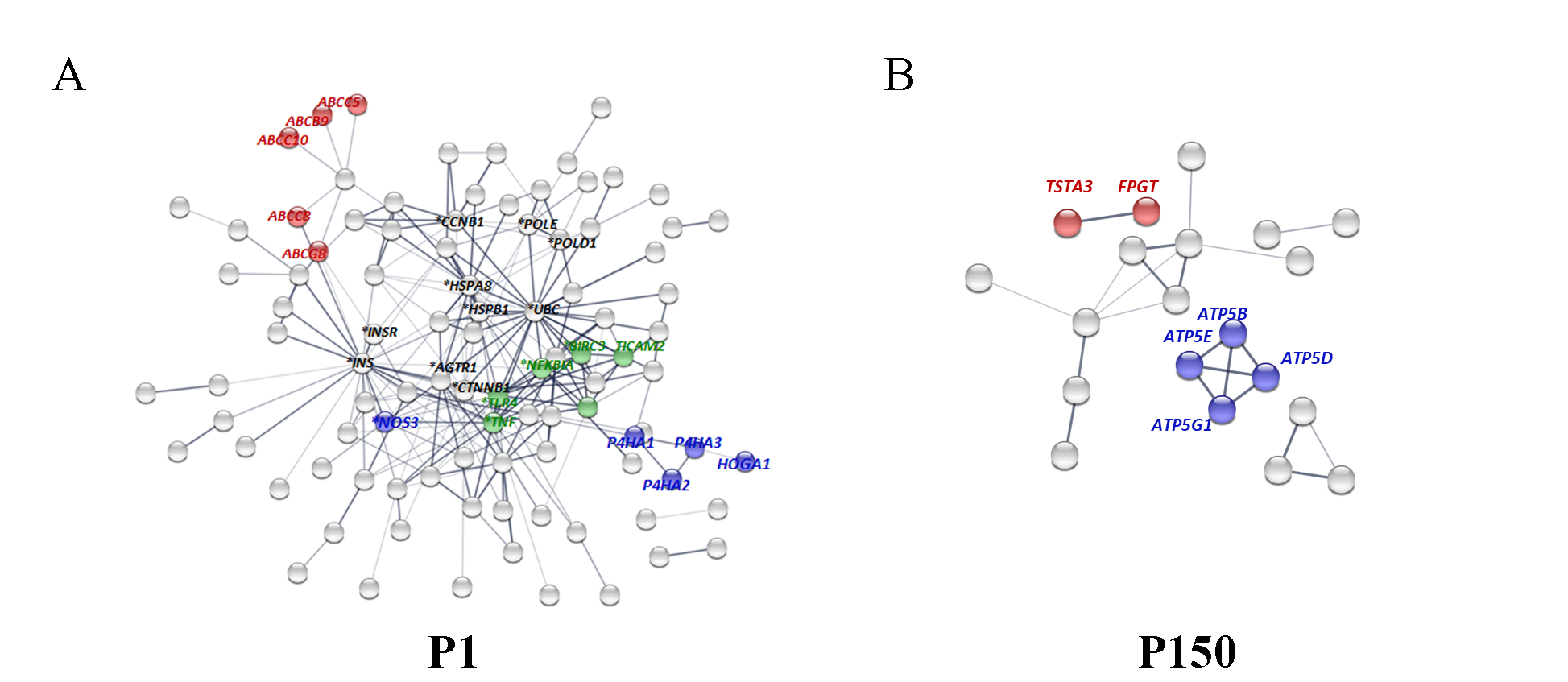

Supplement: Supplementary file 1 [file genes-10-00656-s001.zip › supplement/Figure S4.tif]
